# Mesenchymal Stem Cell Derived Extracellular Vesicles Ameliorate Kidney Injury in Aristolochic Acid Nephropathy

**DOI:** 10.3389/fcell.2020.00188

**Published:** 2020-03-24

**Authors:** Sharad Kholia, Maria Beatriz Herrera Sanchez, Massimo Cedrino, Elli Papadimitriou, Marta Tapparo, Maria Chiara Deregibus, Stefania Bruno, Federica Antico, Maria Felice Brizzi, Peter J. Quesenberry, Giovanni Camussi

**Affiliations:** ^1^Department of Medical Sciences, University of Turin, Turin, Italy; ^2^Molecular Biotechnology Center, University of Turin, Turin, Italy; ^3^2i3T Società per la Gestione dell’Incubatore di Imprese e per il Trasferimento Tecnologico Scarl, University of Turin, Turin, Italy; ^4^FORB, Molecular Biotechnology Centre, University of Turin, Turin, Italy; ^5^Division of Hematology/Oncology, Rhode Island Hospital, Brown University, Providence, RI, United States

**Keywords:** aristolochic acid nephropathy, extracellular vesicles, mesenchymal stem cells, chronic kidney disease, miRNA, fibrosis

## Abstract

Limitations in the current therapeutic strategies for the prevention of progression of chronic kidney disease (CKD) to end stage renal disease has been a drawback to improving patient recovery. It is therefore imperative that a solution is found to alleviate this problem and improve the health and well-being of patients overall. Aristolochic acid (AA) induced nephropathy, a type of nephrotoxic CKD is characterised by cortical tubular injury, inflammation, leading to interstitial fibrosis. Extracellular vesicles derived from human bone marrow mesenchymal stem cells (MSC-EVs) display therapeutic properties in various disease models including kidney injury. In the current study, we intended to investigate the ability of MSC-EVs on ameliorating tubular injury and interstitial fibrosis in a mouse model of aristolochic acid nephropathy (AAN). The chronic model of AAN is comprised of an intraperitoneal injection of AA in NSG mice, followed by a three-day incubation period and then inoculation of MSC-EVs intravenously. This routine was performed on a weekly basis for four consecutive weeks, accompanied by the monitoring of body weight of all mice. Blood and tissue samples were collected post sacrifice. All animals administered with AA developed kidney injury and renal fibrosis. A gradual loss of body weight was observed, together with a deterioration in kidney function. Although no significant recovery was observed in weight loss following treatment with MSC-EVs, a significant reduction in: blood creatinine and blood urea nitrogen (BUN), tubular necrosis, and interstitial fibrosis was observed. In addition, infiltration of CD45 positive immune cells, fibroblasts, and pericytes which were elevated in the interstitium post AA induced injury, were also significantly reduced by MSC-EVs. Kidneys were also subjected to molecular analyses to evaluate the regulation of pro-fibrotic genes. MSC-EVs significantly reduced AA induction of the pro-fibrotic genes α*-Sma*, *Tgfb1* and *Col1a1*. A downregulation in pro-fibrotic genes was also observed in fibroblasts activated by AA injured mTECs *in vitro.* Furthermore, meta-analyses of miRNAs downregulated by MSC-EVs, such as miR21, revealed the regulation of multiple pathways involved in kidney injury including fibrosis, inflammation, and apoptosis. These results therefore suggest that MSC-EVs could play a regenerative and anti-fibrotic role in AAN through the transfer of biologically active cargo that regulates the disease both at a protein and genetic level.

## Introduction

The current limitation in effective therapeutic strategies, available to prevent the progression of chronic kidney disease (CKD) toward end stage renal disease, has been a major drawback in improving patient recovery. It is therefore imperative that a solution is found to alleviate this problem and improve the overall health and well-being of patients (Shea and Booth, 2019).

Aristolochic acids (AA) are a group of toxins that are enriched in plants of the genus *Aristolochia* and *Asarum*, commonly found worldwide (Han et al., 2019). These plants have been regularly used as part of traditional herbal therapy for the treatment of various ailments as well as slimming (Jadot et al., 2017). In the early 1990s, various women who were on a similar weight loss regimen, consisting of Chinese herbs, presented with a rapidly progressive form of CKD, known as Chinese herbal nephropathy (CHN) (Jadot et al., 2017). In addition, another form of nephropathy, more prominent in the Balkan region of Eastern Europe, hence known as Balkan endemic nephropathy (BEN), also exhibited similarities to CHN (Jelakovic et al., 2019). Further investigations led to the discovery of AA in the Chinese herbs, as the cause of CHN. Meanwhile, the contamination of the weed species *Aristolochia clematitis* (rich in AA) in wheat consumed in the Balkan regions was identified to be the cause of BEN (Jadot et al., 2017). As a result, both CHN and BEN are now collectively classified as aristolochic acid nephropathy (AAN) (Jadot et al., 2017). AAN is mainly characterised by tubular damage and atrophy accompanied with interstitial nephritis and extensive fibrosis. In addition, 40% of cases manifest with urothelial carcinoma due to the DNA adducting properties of AA. Although extensive research has been done over the years to elucidate the underlying mechanisms of AAN, no effective therapeutic regimen is available as of yet (Han et al., 2019).

Current advances in the field of regenerative medicine have generated an interest in the development of alternative treatments that would aim not only to alleviate the symptoms, but also to repair and regenerate injured tissues. In particular, therapies based on adult stem cells have become a promising strategy due to their abilities of self-renewal, multipotency, and plasticity. Bone marrow derived mesenchymal stromal cells (MSCs) are one of the most extensively studied stem cell types, as they are easy to isolate/expand, and have a low risk of forming teratomas. In addition, they have shown great therapeutic potential in various experimental models of acute and chronic kidney disease. For instance, Morigi et al. (2004) reported that murine bone marrow derived MSCs in a mouse model of cisplatin-induced acute kidney injury (AKI) alleviated tubular damage and improved renal function. Furthermore, Herrera et al. (2004), in their mouse model of glycerol-induced AKI, also showed a similar tubular regenerative effect attributed to the engraftment of MSCs in the damaged kidney. Apart from AKI, MSCs have also been reported to be therapeutically effective in experimental CKD. For instance, bone marrow derived MSCs attenuated tubulointerstitial injury, in a model of unilateral ureter obstruction in mice (Xing et al., 2019) and promoted renal repair, by limiting the dysfunction of podocytes and glomerular progenitor cells, in a model of adriamycin-induced nephropathy (Zoja et al., 2012). However, due to limitations in the expansion of MSCs *in vitro*, the induction of senescence at an early passage, and immunogenicity, has made the translation of experimental findings to a more clinical setting rather challenging (Rota et al., 2019). Various mechanisms have been suggested through which MSCs exhibit their reno-protective effect. One such mechanism is the packaging and release of biologically active factors in the form of extracellular vesicles (EVs).

Extracellular vesicles are heterogeneous membrane bound particles, naturally released by all cells in the body (Grange et al., 2019a). They are classified according to their biogenesis, whereby, EVs derived from multivesicular bodies are known as exosomes and those released from the cell surface through membrane blebbing are known as ectosomes (van der Pol et al., 2012). EVs influence recipient cells, both locally and systemically, through the transfer of their biologically active cargo (nucleic acids, proteins, and lipids) (Bruno et al., 2019). In addition, they can also induce epigenetic changes in injured target cells, activating regenerative programmes (Derkus et al., 2017).

The regenerative capability of MSC-EVs has been very well described in multiple models of kidney disease. For instance, Bruno et al. (2009) demonstrated that MSC-EVs augmented the recovery of injured tubular cells, by preventing apoptosis and promoting proliferation, in a glycerol induced murine model of AKI. In addition, the effect observed was similar to the effects observed when injecting MSCs in the same model (Bruno et al., 2009). In addition, Alzahrani (2019) showed through a rat model of renal ischemia reperfusion injury, that exosomes derived from MSC alleviate the disease. However, exosomes isolated from melatonin preconditioned MSCs were even more effective in comparison to naïve MSC exosomes. This therefore indicates the potential prospect of enhancing the therapeutic effect of MSC-EVs through preconditioning. MSC-EVs have also proven to be functional in toxic models of AKI, whereby renal function together with the classic pathophysiological features were significantly improved (Bruno et al., 2012; Reis et al., 2012). Apart from AKI, MSC-EVs have also been shown to be effective in various models of CKD. For instance, in a murine model of diabetic nephropathy, Grange et al. (2019b) confirmed the reversion of renal fibrosis and an improvement in renal function post treatment with MSC-EVs. In other models of CKD, such as the 5/6 nephrectomy model and the unilateral ureter obstruction (UUO) model, MSC-EVs have proven to be effective in reducing glomerulosclerosis and fibrosis (He et al., 2012, 2015). Recently, we reported that also human liver stem cell-derived EVs (HLSC-EVs) exhibit regenerative properties and are effective in reducing fibrosis and improving overall kidney function in the current model of AAN (Kholia et al., 2018).

The aim of the current study was therefore to evaluate whether MSC-EVs exhibit any therapeutic effect similar to HLSC-EVs in the CKD model of AAN.

## Materials and Methods

### Cell Culture

#### Mesenchymal Stem Cells (MSC)

Human bone marrow MSCs purchased from Lonza (Basel, Switzerland) were cultured in mesenchymal stem cell basal medium (MSCBM, Lonza). Cells were sub-cultured after 15 days following thawing from passage one and then after every seven days for successive passages. EVs were isolated from cells until passage six.

#### Murine Tubular Epithelial Cells (mTEC)

mTECs, isolated from kidneys of healthy female C57 mice [as described previously by our lab (Bruno et al., 2009)], were cultured in Dulbecco’s modified essential medium (DMEM) supplemented with L-glutamine (5 mM), penicillin (50 IU/ml), streptomycin (50 μg/ml) and 10% FCS (Euroclone, Milan, Italy).

#### Mouse Kidney Cortical Fibroblasts (mkCF)

mkCFs, were isolated from the kidneys of healthy male CD1 mice [as previously described by our lab (Kholia et al., 2018)], were cultured in DMEM high glucose (Euroclone, Milan, Italy) supplemented with L-glutamine (5 mM), penicillin (50 IU/ml), streptomycin (50 μg/ml), 10 ml HEPES (Sigma Aldrich), and 10% FCS (Euroclone, Milan, Italy).

### Isolation and Characterisation of EVs

EVs were isolated from supernatants, obtained from MSCs (1 × 10^6^ cells/T150 flask) following overnight (18 h) starvation in serum free Roswell Park Memorial Institute Medium (RPMI) (Euroclone, Milan, Italy). The viability of cells post starvation was 98%, as confirmed by Trypan blue exclusion staining. Briefly, supernatants were subjected to centrifugation at 3000 *g* for 15 min at 4°C, followed by microfiltration using a 0.22 μm vacuum filter unit (Millipore, United States), for the removal of cell debris and apoptotic bodies. The effective removal of cell debris by this procedure was checked by electron microscopy. In order to pellet EVs, the purified supernatant was further ultracentrifuged at 100,000 *g* for 2 h at 4°C using the SW70Ti rotor in a Beckman Coulter Optima L-90 K ultracentrifuge (Beckman Coulter, Fullerton, CA, United States). The resulting pellet was resuspended in RPMI supplemented with 1% dimethyl sulfoxide (DMSO) and stored at -80°C.

Characterisation of EVs was performed according to the criteria suggested by the ISEV position paper (Thery et al., 2018). The expression of surface markers was evaluated using the human cytofluorimetric bead-based MACSPlex exosome kit (Miltenyi Biotec, Germany) according to manufacturer’s protocol. Briefly, three independent MSC-EV preparations consisting of approximately 1 × 10^9^ MSC-EVs/preparation were diluted in MACSPlex buffer (MPB) to a final volume of 120 μl in a 1.5 ml microcentrifuge tube. This was followed by the addition of 15 μl of MACSPlex exosome capture beads (containing a cocktail of 39 different exosomal marker epitopes). The EVs on capture beads were counterstained by adding 5 μl of APC-conjugated: anti-CD9, anti-CD63, and anti-CD81 detection antibodies to each of the tubes and incubating for 1 h at room temperature in the dark on an orbital shaker at 450 rpm. Post incubation, the beads were subjected to first washing with 1 ml of MPB at 3,000 *g* for 5 min, followed by a longer washing step by incubating the beads in 1 ml of MPB on an orbital shaker (as before) for 15 min. After this step, the beads were centrifuged at 3,000 *g* for 5 min and the supernatant was carefully aspirated leaving a residual volume of 150 μl per tube for acquisition. Flow cytometric analysis was performed using the Cytoflex flow cytometer (Beckman Coulter, Brea, CA, United States) whereby approximately 5000–8000 single bead events were recorded per sample. The median fluorescence intensity (MFI) for all 39 exosomal markers were corrected for background and gated based on their respective fluorescence intensity as per manufacturer’s instructions.

Transmission electron microscopy was performed as described previously (Kholia et al., 2018) using negative staining with NanoVan (Nanoprobes, Yaphank). Samples were examined by a Jeol JEM 1010 electron microscope (Joel, United States). Electron microscopy revealed the presence of vesicles ranging from 35 to 100 nm. Western blot analysis of EV protein was performed to confirm the presence of the classical exosomal marker CD63, and the absence of the cytoplasmic marker GM130 (positive in MSC cell lysates). Particle size and concentration of purified EVs was assessed with the Nanosight NS300 (NanoSight, Amesbury, United Kingdom) equipped with a 405 nm laser using the NTA 1.4 Analytical Software as described previously (Herrera Sanchez et al., 2014).

### *In vitro* Model of Aristolochic Acid Nephropathy

In order to elucidate the effects of MSC-EVs on cortical renal fibroblasts, an *in vitro* model of AA-induced fibrosis was set. Briefly, 1.5 × 10^4^ mTECs were treated with 100 μM AA for 4 h in 24 well cell culture inserts (1.0 μm pore) (Thermo Fisher Scientific). mTECs without AA treatment served as controls. Post incubation, the mTECs were washed once with PBS and co-cultured with mkCF cells (already seeded the day before in a 24 well plate at a concentration of 2 × 10^4^ cells/well) for five days at 37°C in the presence or absence of MSC-EVs at a concentration of 75,000 EVs/cell (MSC-EVs were added to the lower compartment with fibroblasts). Post incubation, mkCFs were analysed for fibrotic gene expression by qRT-PCR. Fibroblasts co-cultured with healthy mTECs served as controls.

### Mouse Model of Aristolochic Acid Induced Nephropathy

Animal studies were conducted according to the guidelines for the Care and Use of Laboratory Animals set by the National Institute of Health. All procedures were approved by the Ethics Committee of the University of Turin and the Italian Health Ministry (authorisation number: 766/2016-PR). AAN was instigated in male NOD/SCID/IL2Rγ KO (NSG) mice (bred at the animal facility in the Molecular Biotechnology Centre) (6/8 weeks old; *n* = 12) by intraperitoneal injections of 4 mg/kg of AA diluted in 250 μl of PBS final volume (Santa Cruz Biotechnology, Santa Cruz, CA, United States) (AA was dissolved in DMSO at a concentration of 10 mg/ml) once a week for four consecutive weeks (Figure 2A). A group of mice were injected with MSC-EVs (*n* = 9) intravenously at a concentration of 1 × 10^10^ EVs/ml/mouse or vehicle alone (PBS) (*n* = 5; as control) three days after AA administration on a weekly basis (Figure 2A). Mice were sacrificed after four weeks and subjected to multi-parameter analyses as mentioned below. The use of immunodeficient mice was preferred in this study to avoid an immunogenic reaction due to repeated injections of MSC-EVs. During AA administration, healthy mice were injected with the volume of DMSO (equivalent to 4 mg/kg of AA) diluted in 250 μl of PBS final volume.

### Total Body Weight and Kidney Function

Body weight of mice was recorded to assess their general health on a weekly basis prior to AA injections and at the end of the experiment just before sacrificing. Kidney function of mice from all experimental groups was evaluated by measuring blood plasma creatinine after sacrificing using a colorimetric microplate assay based on the Jaffe reaction (Quantichrom Creatinine Assay, BioAssay systems, Hayward, CA, United States) as per manufacturer’s protocol. Blood urea nitrogen (BUN) was measured by direct quantification of plasma urea with a colorimetric assay kit according to the manufacturer’s protocol (Quantichrom Urea Assay, BioAssay Systems, Hayward, CA, United States).

### RNA Extraction and qRT-PCR

Total RNA was extracted from mouse tissue as described previously (Kholia et al., 2018). Briefly, mouse renal tissue was resuspended in 1 ml of TRIzol^TM^ solution (Ambion, Thermofisher) in microcentrifuge tubes and homogenised in a Bullet blender (Next Advance Inc, NY, United States) at a speed of 8 rpm for 3 min using 3.2 mm size zirconium beads. The tubes were then placed on a tube rotator at 4°C for 30 min, followed by centrifugation at 12,000 *g* for 15 min at 4°C. Supernatant was transferred to clean tubes and subjected to RNA isolation using the miRNeasy mini kit (Qiagen, Frederick, MD, United States), according to the manufacturer’s protocol. For the isolation of RNA from cells, 700 μl of TRIzol^TM^ solution was added to cells in the wells and incubated for 10 min on a shaker. The mixture was then transferred to 1.5 ml RNAse free tubes and subjected to RNA isolation using the miRNeasy mini kit (Qiagen, Frederick, MD, United States), according to the manufacturer’s protocol. Total RNA was quantified using the NanoDrop2000 spectrophotometer (Thermo Fisher, Waltham, MA, United States) and either used immediately or stored at -80°C until further use.

cDNA was synthesised by retro-transcribing 200 ng of total RNA using the High Capacity cDNA reverse transcription kit (Thermo Fisher, Waltham, MA, United States), according to the manufacturer’s protocol. qRT PCR was performed using the StepOnePlus RT-PCR machine (Thermo Fisher, Waltham, MA, United States) in 20 μl reactions with Power SYBR Green PCR Master Mix (Thermo Fisher, Waltham, MA, United States) and specific oligonucleotide primers (Table 1) (MWG−Biotech, Eurofins Scientific, Brussels, Belgium). Data were analysed using ΔΔCt method with *Gapdh* as endogenous control.

**TABLE 1:** List of Primers used for qRT-PCR.

**Gene**	**Primer Sequence 5′-3′**
m_*Col1a1* Forward	ATC TCC TGG TGC TGA TGG AC
m_*Col1a1* Reverse	ACC TTG TTT GCC AGG TTC AC
m_*Tgfb1* Forward	CGA AAG CCC TGT ATT CCG TCT
m_*Tgfb1* Reverse	GCA ACA ATT CCTGGC GTT ACC
m_α*-Sma* Forward	CTG ACA GAG GCA CCA CTG AA
m_α*-Sma* Reverse	CAT CTC CAG AGT CCA GCA CA
m_*Ltbp1* Forward	GGA GCC CGA AGT GGT AAC AG
m_*Ltbp1* Reverse	GAA TAG TTG AAA CCC CTG GGG
m_*Gapdh* Forward	TGT CAA GCT CAT TTC CTG GTA TGA
m_*Gapdh* Reverse	TCT TAC TCC TTG GAG GCC ATG T
hsa-miR-132-5p	TAA CAG TCT ACA GCC ATG GTC G
hsa-miR-342-3p	TCT CAC ACA GAA ATC GCA
hsa-miR-214-3p	ACA GCA GGC ACA GAC AGG
hsa-RNU6b	CGC AAG GAT GAC ACG CAA

Validation of the expression of specific miRNAs obtained from the Fireplex^®^ assay by ABCAM, was performed as follows. Total RNA was isolated from ten (8 μm) formalin fixed parafilm embedded (FFPE) sections (*n* = 3/condition) using the RecoverALL^TM^ Total Nucleic acid isolation kit for FFPE (Thermo Fisher, Waltham, MA, United States) according to the manufacturer’s protocol. The total RNA from each mouse (200 ng of input RNA) was reverse transcribed, using the miScript Reverse Transcription Kit (Thermo Fisher, Waltham, MA, United States) and the cDNA was subjected to RT-PCR, to validate the miRNAs of interest. Experiments were performed in triplicate using 3 ng of cDNA for each reaction as described by the manufacturer’s protocol (Qiagen). The following miRNAs were screened in all mice conditions: miR-132-5p, miR-342-3p, and miR-214-3p with RNU6b as endogenous control.

### Histological Analysis

Paraffin embedded renal tissues were cut in 5 μm-thick sections and stained with haematoxylin and eosin (for tubular damage and hyaline cast formation), or Masson’s Trichrome (for interstitial fibrosis) (Bio-Optica, Milan, Italy), according to the manufacturer’s protocols. Assessment of tubular necrosis was performed by analysing 10 non-overlapping cortical fields/section at a magnification of 400x (high power field, HPF) using ImageJ software. Quantification of interstitial fibrosis was performed by measuring collagenous fibrotic areas stained in blue (sections stained with Masson’s trichrome) in 10 random cortical fields/section from images taken at a magnification of 200x, using multiphase image analysis with ImageJ software version 1.49s (Schneider et al., 2012).

Immunohistochemical staining was performed as described previously (Herrera Sanchez et al., 2014). Briefly, 5 μm sections were deparaffinised, hydrated, and subjected to antigen-retrieval. Endogenous peroxidase was removed using 5% H_2_O_2_. Sections were blocked with 3% BSA/PBS and then incubated with antibodies of interest: proliferating cell nuclear antigen (PCNA) (1:400, Santa Cruz Biotechnology), α-smooth muscle actin (α-SMA) (1:100, Ab7817, Abcam), S100A4 [Fibroblast specific protein 1 (FSP-1)] (1:500, Ab41532, Abcam), and CD45 (1:500, Ab10558, Abcam) overnight at 4°C. The following day, after two washes with PBS-tween 0.01%, the sections were incubated with secondary horse radish peroxidase antibody (Pierce, Rockford, IL, United States) for 1 h at room temperature. Sections were then developed with diaminobenzidine (DAB) (Dako, Carpinteria, CA, United States), counterstained with haematoxylin, and analysed via microscopy.

For sections that required immunofluorescence double staining, the slides were blocked with 3% BSA/PBS for 30 min following antigen retrieval, permeabilised in 0.2% Triton-X100/PBS for 6 min at 4°C and then incubated with the primary antibodies of interest: α-SMA (1:100, Ab7817, Abcam), and platelet derived growth factor receptor beta (PDGFRβ, 1:50, Ab32570, Abcam) overnight at 4°C. Secondary antibodies were incubated with sections for 1 h at room temperature. Following washes, the sections were stained with DAPI and mounted with Fluorescent mounting media and analysed via microscopy. Sections labelled only with secondary antibody served as controls.

In order to evaluate changes in cell death, kidney tissue sections were stained for DNA fragmentation using terminal deoxynucleotidyl transferase dUTP nick end labeling (TUNEL) according to the manufacturer’s protocol (Roche, United Kingdom). Cell death was quantified by averaging the numerical count of TUNEL-positive nuclei in 8 random cortical fields/section from images taken at a high magnification of 400x, using ImageJ software as mentioned above.

### Fireplex miRNA Assay

The FirePlex multiplex miRNA allows a high-throughput analysis of up to 65 literature validated peer-reviewed miRNA targets, involved in kidney injury, by flow cytometry and efficient analysis with the FirePlex Analysis Workbench Software. In order to evaluate the regulation of miRNAs specifically involved in kidney injury, formalin fixed paraffin embedded tissue slices (in duplicate) from mice treated with AA (*n* = 3), AA mice treated with MSC-EVs (*n* = 5), and healthy mice (*n* = 3) were provided to Abcam to perform the assay specific to kidney injury (kidney toxicity panel, ab219508, Abcam) as a service. The samples were processed as per the published protocol. Briefly, FFPE samples were mixed with 36 μl Digest Buffer, 20 μl water and 4 μl Protease Mix and incubated at 60°C for 45 min with shaking. For each sample run, FirePlex Particles (35 μl) were added to a well of a 96-well filter plate and filtered. Twenty-five μl Hybe Buffer was added to each well followed by 1 ng of total RNA. The plate was incubated at 37°C for 1 h with shaking. After two washes with 1 × Rinse A, 75 μl of 1× Labeling Buffer was added per well. The plate was further incubated at room temperature for 1 h with shaking. After two washes with 1 × Rinse B and one wash with 1 × Rinse A, a catch plate was added to the vacuum manifold and the filter plate put under constant vacuum. Sixty five μl of 95°C RNAse-free water was added twice to each well to elute the ligated sample. Thirty μl of this meltoff was added to a clean PCR plate and mixed with 20 μl PCR master mix. The mixture underwent 32 cycles of PCR amplification. Sixty μl of Hybe Buffer was then added back to each well of the original particles followed by 20 μl of the PCR product, and the plate was incubated at 37°C for 30 min with shaking. After two washes with 1 × Rinse B and one with 1 × Rinse A, 75 μl of 1 × Reporting Buffer was added per well and the plate was incubated at room temperature for 15 min on a shaker. Post incubation, the plate was washed twice with 1 × Rinse A, and then 175 μl of Run Buffer was added to each well. The samples were then scanned on an EMD Millipore Guava 6HT flow cytometer (MilliporeSigma, Germany) and the Flow cytometry quantification data was analysed with the FirePlex Analysis Workbench software (Abcam, Cambridge, MA, United States) whereby, the Fluorescence intensity values across all samples were normalised using the geNorm algorithm.

### Bioinformatic Analyses

Data from the Fireplex miRNA assay were further analysed using FirePlex^®^ Analysis Workbench Software version 2.0 (Abcam) Fireplex Analysis Workbench software (Abcam). Gene target prediction was performed with miRWalk 3.0 considering only miRNAs targeting the 3′UTR sequence and a score above 0.95 (Dweep et al., 2014). Panther Classification System online software was used for pathway enrichment analysis (Thomas et al., 2003). Only pathways with a minimum number of five genes were considered in the classification.

### Statistical Analyses

All data obtained were analysed using GraphPad Prism 6.0. Results are expressed as mean ± standard deviation (SD) or standard error of the mean (SEM) where indicated. Statistical analyses were performed by employing the one way analyses of variance (ANOVA) or two-way ANOVA with a multi comparison test where appropriate. A *p*-value of <0.05 was considered statistically significant.

## Results

### Characterisation of MSC-EVs

**FIGURE 1:**
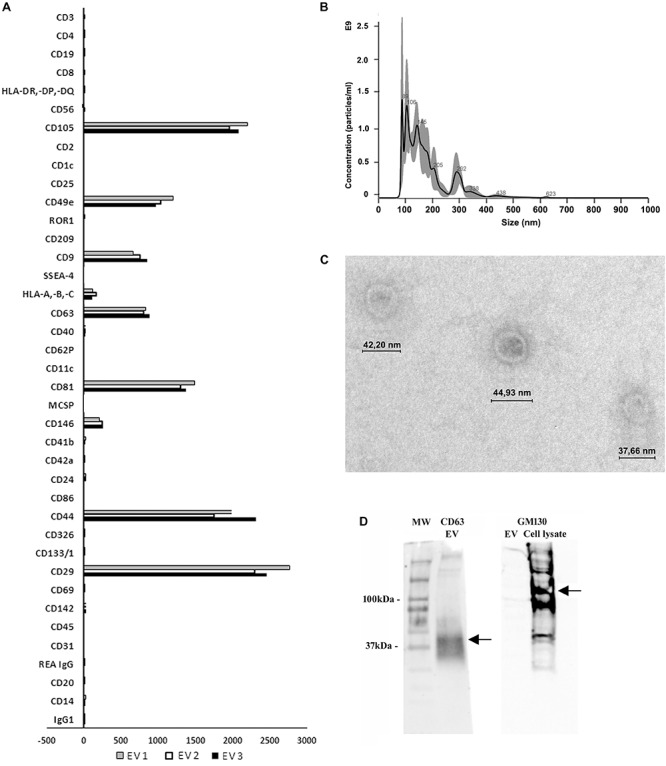
Characterisation of MSC-EVs. **(A)** Multiplex bead-based flow cytometry assay was used to characterise MSC-EV surface antigens. Thirty nine multiplexed populations of dye-labeled antibody-coated capture beads containing established exosomal markers were incubated with MSC-EVs and analysed by flow cytometry. Experiments were performed with three different samples. **(B)** Nanoparticle tracking analyses showing the size distribution and quantity of MSC-EVs purified by differential ultracentrifugation. **(C)** Representative transmission electron microscopy showing MSC-EVs (original magnification 150,000X). **(D)** Representative Western blot analysis of the exosomal marker CD63 (30–60 kDa) in MSC-EVs and negatively stained for GM130 (130 kDa) (MSC lysate was used as positive control).

EVs were evaluated for typical expression markers using the MACS multiplex bead-based flow cytometry assay described recently (Koliha et al., 2016; Wiklander et al., 2018). The assay involves incubating EVs with a cocktail of 39 different bead populations, each representing a typical exosomal surface antigen (37 markers and 2 isotypic controls). EVs bound to beads are counterstained with a cocktail of APC conjugated detection antibodies of commonly used exosome markers anti-CD9, anti-CD63, and anti-CD81 and analysed by flow cytometry. Data analysis revealed that EVs were positive for the typical MSC markers such as CD29, CD44, CD49e, CD105, CD146, as well as for the exosomal positive tetraspanins CD9, CD63, and CD81 (Figure 1A). In addition, EVs were negative for the endothelial marker CD31 and epithelial marker CD326, therefore confirming their mesenchymal origin (Figure 1A). Furthermore, Nanosight analysis revealed the size of EVs to be in the typical range of 30-300 nm, which was further confirmed by transmission electron microscopy (Figures 1B,C). Western blot analysis showed that EVs were positive for CD63 and negative for the cell cytoplasmic marker GM130 (Figure 1D).

### MSC-EVs Ameliorate AA Induced Kidney Damage

**FIGURE 2:**
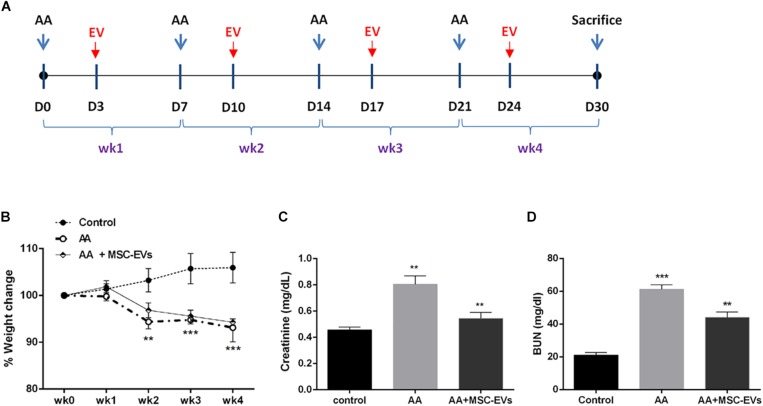
Induction of nephropathy in NSG mice by aristolochic acid intoxication. **(A)** Schematic representation of the experimental design for aristolochic acid nephropathy (AAN) *in vivo* showing the days of administration of AA or Human bone marrow mesenchymal stem cell-derived extracellular vesicles (MSC-EVs) for 4 weeks (wk). **(B)** Mice body weight was measured weekly before AA injections for 4 weeks. The body weight is represented as percentage weight change. A two-way analyses of variance (ANOVA) was performed with Bonferroni’s multi comparison test; Data are expressed as mean ± SEM of seven mice per group. **p and ***p < 0.001 AA vs. control. **(C)** Plasma creatinine and **(D)** BUN levels in mice treated with vehicle alone or mice injected with AA or AA mice injected with MSC-EVs (*n* = 7 mice per group). **p < 0.001 AA vs. control and AA + MSC-EVs vs. AA. Data are expressed as mean ± SEM; a one way ANOVA with Bonferroni’s multi comparison test was performed.

Weight loss is considered to be one of the main macro symptoms of AAN. Therefore, we monitored the total body weight of each mouse on a weekly basis prior to AA administration (Figure 2A). Mice injected with AA lost weight significantly from week two onward compared to healthy mice (Figure 2B). Treatment with MSC-EVs, post AA administration, did not ameliorate the loss of weight induced by AA throughout the course of the pathology (Figure 2B). As a parameter of kidney function, the plasma creatinine and BUN levels were also evaluated in all mice groups at sacrifice. Data analysis revealed a significant increase in plasma creatinine and BUN levels of mice injected with AA compared to healthy controls (Figures 2C,D). In contrast, AA mice treated with MSC-EVs had significantly reduced levels of both plasma creatinine and BUN in comparison with mice injected with AA alone (Figures 2C,D).

**FIGURE 3:**
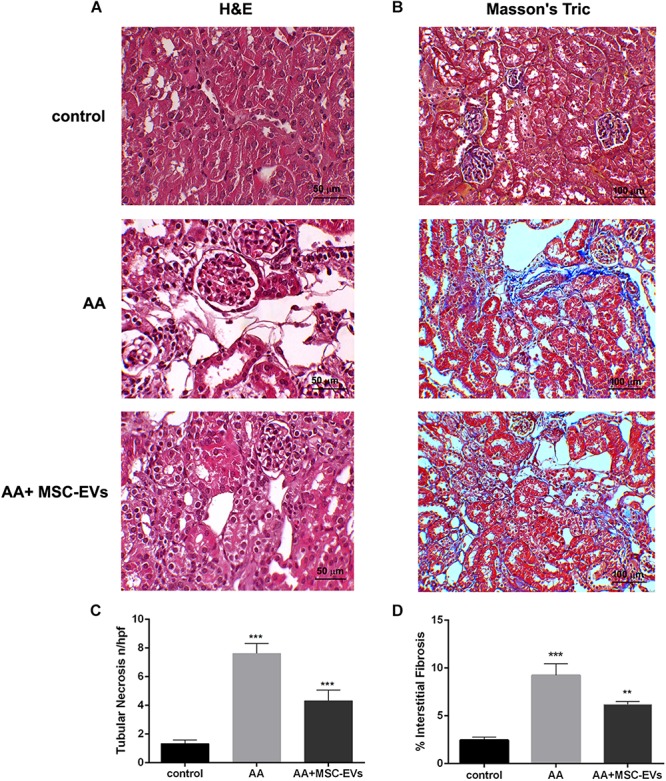
Histological analyses of AAN *in vivo* model. **(A)** Micrographs representing H&E stained renal tissue from healthy mice injected with the vehicle alone (control), or mice injected with AA, or AA mice treated with MSC−EVs. Original magnification at 400X. **(B)** Micrographs representing Masson’s trichrome stained renal sections from control, AA or MSC-EV treated AA mice. The blue stain is collagen fibres signifying interstitial fibrosis. Original magnification at 200X. **(C)** Histological score of tubular necrosis in AAN mice experimental groups. Mice treated with AA had significantly high levels of tubular necrosis, which was reduced, on treatment with MSC-EVs. Data represent mean ± SEM of tubular necrosis observed under high power field (original magnification: 400X). A one way ANOVA with Bonferroni’s multi comparison test was performed. ***p < 0.001 AA vs. control or AA+MSC−EVs vs. AA. **(D)** Histological quantification of interstitial fibrosis in AAN mice experimental groups by multiphase image analysis of 10 fields per section. Data represent mean ± SEM; A one way ANOVA with Bonferroni’s multi comparison test was performed. ***p < 0.001 AA vs. control, **p < 0.01 MSC−EV vs. AA.

**FIGURE 4:**
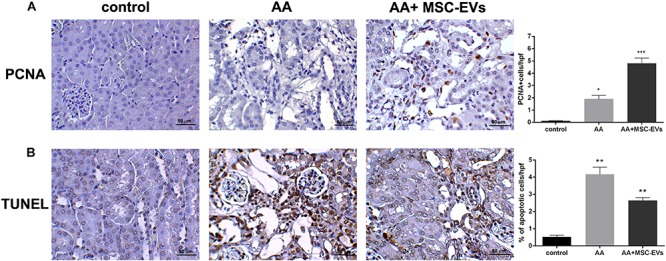
Representative micrographs of PCNA and TUNEL stained renal tissue. **(A)** Proliferating cell nuclear antigen (PCNA) staining of control, AA, or AA mice treated with MSC-EVs. Histological score of PCNA positive cells in AAN mice experimental groups observed under high power with an original magnification of 400X. An increase in PCNA positive cells was observed in MSC-EV treated mice renal tissue. Data represent mean ± SEM. A one way ANOVA with Bonferroni’s multi comparison test was performed. *p < 0.01 AA vs. control, or ***p < 0.05 AA+MSC−EVs vs. AA **(B)** TUNEL staining of control, AA, or AA mice treated with MSC-EVs. Histological score of% of apoptotic cells (by TUNEL staining) in AAN mice experimental groups observed under high power with an original magnification of 400X. A decrease in TUNEL positive cells was observed in MSC-EV treated mice renal tissue. Data represent mean ± SEM. A one way ANOVA with Bonferroni’s multi comparison test was performed. **p < 0.01 AA vs. control, or **p < 0.05 AA+MSC−EVs vs. AA. Original magnification at 400X.

Immunohistochemical analyses were performed to evaluate the morphological changes of kidney tissue from all the experimental groups. Kidney tissue of mice that had been injected with AA alone had severe damage of the proximal tubules, formation of hyaline casts, and development of interstitial fibrosis, compared to healthy controls (Figures 3A,B). On the other hand, mice that received treatment with MSC-EVs showed a significant amelioration in kidney damage, whereby there was a marked reduction in tubular necrosis (H&E staining) and interstitial fibrosis, as observed by Masson’s trichrome staining (Figures 3A,B). This was further confirmed by the histological score (obtained by quantifying tubular necrosis and interstitial fibrosis in kidney sections from all experimental groups) whereby, mice inoculated with MSC-EVs significantly reduced the number of necrotic tubules, and interstitial fibrosis compared to AA injured mice (Figures 3C,D). Furthermore, assessment of proliferation and apoptosis through PCNA/TUNEL staining showed a significant increase in PCNA positive cells (Figure 4A) and a decrease in TUNEL positive apoptotic cells (Figure 4B) in AA mice treated with MSC-EVs compared to mice injected with AA alone.

**FIGURE 5:**
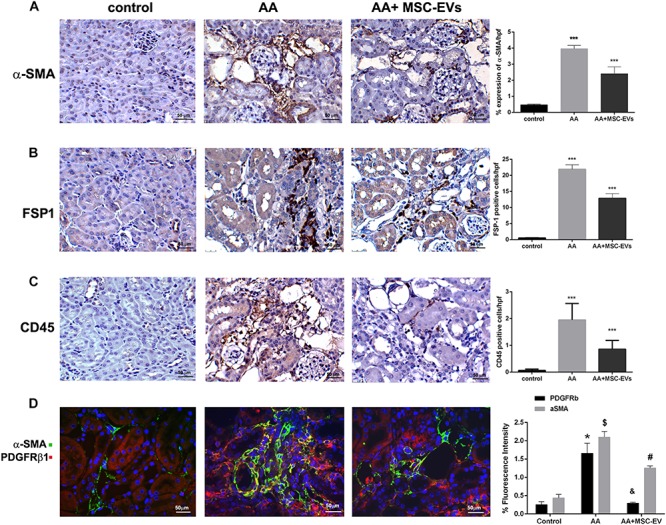
Immunohistological staining of kidneys from mice treated with AA. Kidney paraffin-sections from healthy mice, or mice treated with AA, or AA mice treated with MSC-EVs were stained for α-SMA **(A)**, FSP-1 **(B),** or CD45 **(C)** to identify presence of active fibroblasts and inflammatory cell infiltration (original magnification: 400X). **(D)** PDGFRβ/α-SMA staining was also performed to identify Pericyte/myofibroblast transition in the renal interstitium of kidney paraffin-sections from AAN mice experimental groups (original magnification: 400X). The histograms represent the quantification of cells positive for the relative immunostaining in mouse kidney paraffin-sections from AAN mice experimental groups. Data represent mean ± SEM of the fluorescence intensity of cells positive per high power field measured from 10 images taken at random from six samples per treatment. ^∗∗∗^*p* < 0.001 AA vs. control or AA+MSC−EVs vs. AA. A one way ANOVA with Bonferroni’s multi comparison test was performed. ^∗^ and $: AA vs. Control, *p* < 0.001; & and #: AA+MSC−EV vs. AA, *p* < 0.001.

Activation of fibrogenic cells, as assessed by α-SMA staining, was found to be significantly elevated in mice injected with AA compared to healthy controls (Figure 5A). Interestingly, this elevation was significantly reduced in AA mice treated with MSC-EVs as evaluated by morphometric analyses (Figure 5A). Infiltration of FSP-1 positive cells, and CD45 positive inflammatory cells was also assessed through staining with their respective antibodies. Mice treated with AA had a significantly higher presence of both FSP-1 and CD45 cells compared to healthy controls (Figures 5B,C). In contrast, treatment of AA mice with MSC-EVs significantly reduced the infiltration of FSP-1 and CD45 positive cells (Figures 5B,C). This was further confirmed by histological score analyses which revealed a significant difference between the three experimental groups, exhibiting an ameliorating effect of MSC-EVs (Figures 5B,C). Pericytes are perivascular cells that migrate to the interstitium and differentiate into myofibroblasts during renal interstitial fibrosis. The presence of pericytes/myofibroblasts was therefore assessed through PDGFrβ/α-SMA co-staining. Data analyses revealed a significant rise in PDGFrβ/α-SMA positive cells in the kidney interstitium following AA induced injury compared to healthy controls (Figure 5D). However, AA mice treated with MSC-EVs had significantly lower levels of PDGFrβ/α-SMA positive cells compared to AA mice (Figure 5D).

### MSC-EVs Revert AA Induced Upregulation of Pro-fibrotic Genes in AAN Mice Kidneys

**FIGURE 6:**
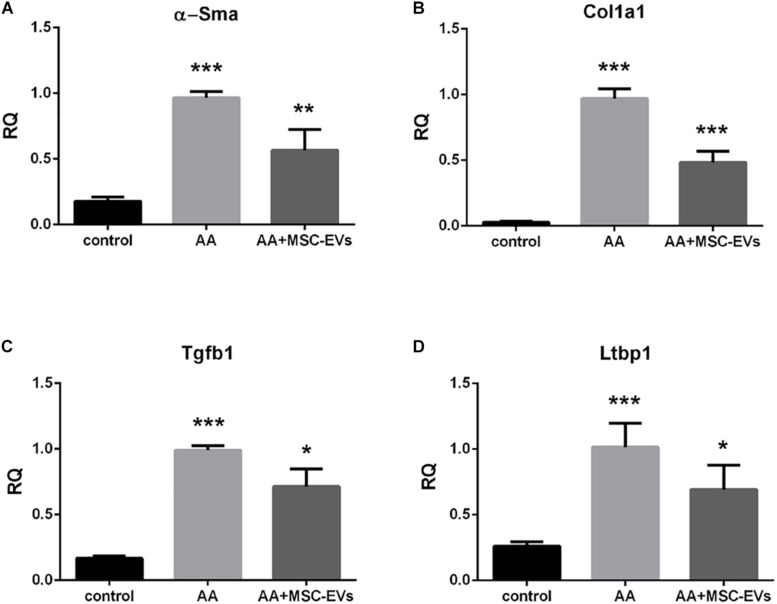
MSC-EVs downregulate pro-fibrotic genes in mice treated with AA. Gene expression levels of α*-Sma* **(A)**, *Col1a1* **(B)**, *Tgfb1* **(C),** and *Ltbp1* **(D)** in mice treated with vehicle alone (control), or mice treated with AA, or AA mice treated with MSC-EVs. Data show mean ± SEM of *n* = 7 samples per treatment. ^∗∗∗^*p* < 0.001 AA vs. control, ^∗^*p* < 0.05, ^∗∗^*p* < 0.01, ^∗∗∗^*p* < 0.001 AA+MSC−EVs vs. AA. A one way ANOVA with Bonferroni’s multi comparison test was performed.

Real time PCR of kidney tissue from the experimental mice groups revealed that mice treated with AA alone had significantly elevated levels of the pro-fibrotic genes: alpha Smooth muscle actin (α*-Sma*), Collagen 1a1 (*Col1a1*) and Transforming Growth Factor beta 1 (*Tgfb1*) (Figures 6A–C) compared to healthy controls. However, AA mice, that had received MSC-EVs, exhibited significantly lower levels of all three pro-fibrotic genes (Figures 6A–C) in comparison with AA injured mice. Furthermore, we also evaluated the regulation of the gene Latent-Transforming Growth Factor beta-binding protein 1 (*Ltbp1*), that codes for the LTBP1 protein, involved in the activation of TGFβ1. Mice treated with AA had significantly upregulated levels of the *Ltbp1* gene which was found to be significantly downregulated in AA mice treated with MSC-EVs (Figure 6D).

### MSC-EV Treatment Induced the Downregulation of Pro-fibrotic Genes in Fibroblasts *in vitro*

**FIGURE 7:**
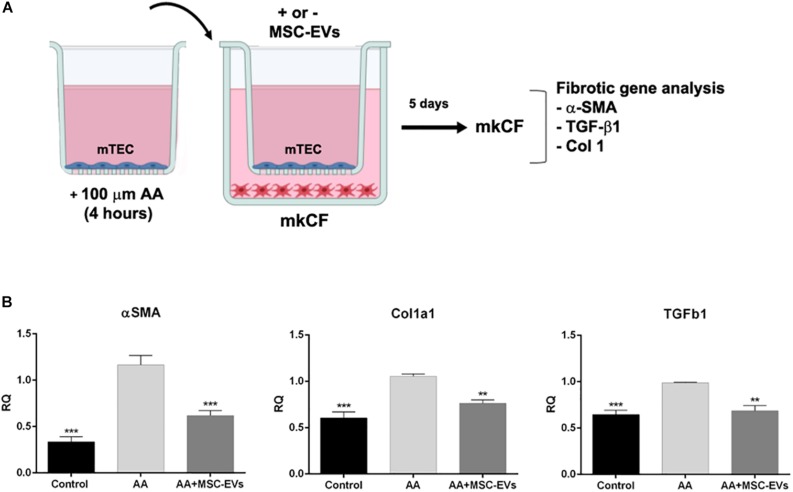
MSC-EVs downregulate pro-fibrotic genes in kidney cortical fibroblasts in an *in vitro* model of AAN. **(A)** An illustration depicting the AAN *in vitro* assay whereby: mTECs treated with or without AA for 4 hrs were co-cultured with mouse fibroblasts (mkCF) in the presence or absence of MSC-EVs (75,000 EVs/cell) for 5 days at 37°C. **(B)** Gene expression analyses revealed an upregulation of the profibrotic markers: α*-Sma*, *Tgfb1*, and *Col1a1* in mkCFs co-cultured with AA treated mTECs. The expression levels of all three pro-fibrotic genes were reduced significantly following treatment with MSC-EVs. The data represents the mean ± SEM of three independent experiments performed in quadruplicate. **p < 0.01 vs. AA, ***p < 0.001 vs. AA. A one way ANOVA with Bonferroni’s multi comparison test was performed.

In order to elucidate the role of MSC-EVs on activated renal fibroblasts, an *in vitro* model in which AA injured mTECs were co-cultured with kidney fibroblasts in a transwell system was set-up (Figure 7A). Analyses of pro-fibrotic genes in renal fibroblasts revealed a significant increase in α*-Sma, Tgfb1*, and *Col1a1* expression in fibroblasts exposed to mTECs pre-treated with AA (Figure 7B). Following treatment with MSC-EVs, this upregulation of pro-fibrotic genes was significantly reduced (Figure 7B), therefore confirming the direct effect of MSC-EVs on activated fibroblasts.

### MSC-EVs Reverse the Expression of miRNAs Dysregulated in AAN

**FIGURE 8:**
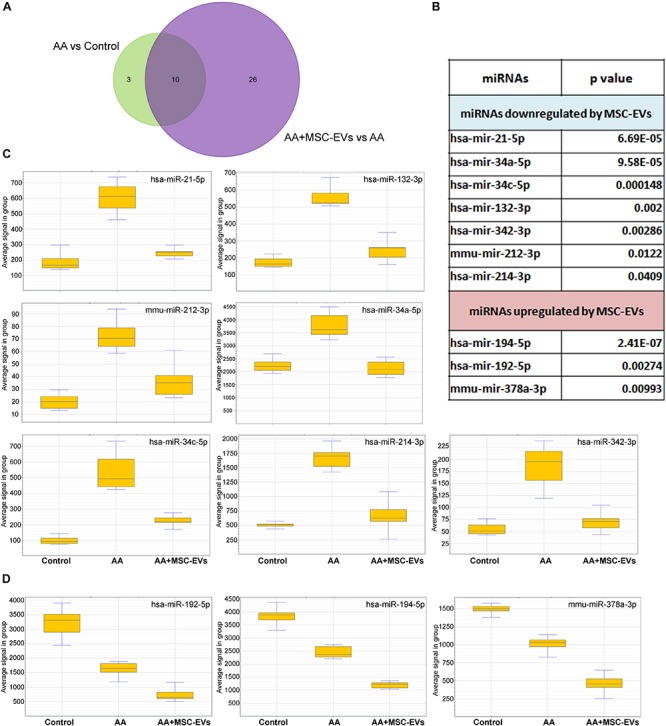
FirePlex miRNA assay. miRNAs, regulated in kidney toxicity, were detected in paraffin kidney sections in mice from all experimental groups (*n* = 3 per group). **(A)** Venn diagram comparing the miRNAs regulated by AA and by MSC-EVs in the AAN model. Thirteen miRNAs were dysregulated in AA intoxicated mice with respect to control mice and 36 in AA mice treated with MSC-EVs. Ten miRNAs were found to be common between the two groups. **(B)** List of the 10 modified miRNAs common between AA mice and AA mice treated with MSC-EVs. **(C)** Box plots representing the miRNAs that were upregulated in mice injured with AA and downregulated following treatment with MSC-EVs. **(D)** Box plots representing the miRNAs that were common between AA mice and AA mice treated with MSC-EVs but not downregulated.

miRNAs have been reported to play a crucial role in the regulation of genes involved in kidney injury (Chung and Lan, 2015). Furthermore, it has also been very well established that EVs can influence the regulation of nucleic acids such as miRNAs by transduction or horizontal transfer to recipient cells or tissue (Ratajczak et al., 2006; Deregibus et al., 2007; Valadi et al., 2007). The regulation of miRNAs by MSC-EVs, following AA injury in mice, was evaluated using the mouse Fireplex miRNA assay. Out of the 65 miRNAs analysed, 13 were found to be significantly dysregulated in mice injured with AA and 36 were found to be significantly regulated following treatment with MSC-EVs (Figure 8A) (Supplementary Tables S1, S2). The comparison of miRNAs between AA and AA + MSC-EV groups, revealed ten to be commonly regulated. Among them, seven were significantly downregulated following MSC-EV treatment (Figures 8B,C), whereas three miRNAs were upregulated with respect to AA treated mice and healthy controls (Figures 8B,D). Three out of the seven miRNAs were validated through RT-PCR (Supplementary Figure S1).

We further analysed the seven miRNAs (Figure 8C) downregulated by MSC-EVs to identify the genes and pathways regulated by them. The list of predicted target genes identified through Mirwalk (data not shown), was further analysed via Panther gene ontology software online. Ninety nine pathways were predicted to be linked to miRNA targeted genes (Supplementary Table S3) out of which the top 37 pathways were selected based on a cut off of >5 genes/pathway and the relevance of the pathway in kidney injury (Table 2). Furthermore, analyses on validated pathways revealed the regulation of 14 pathways (Table 3). Interestingly, the majority of pathways identified have been implicated in various pathological processes such as fibrosis, inflammation, and apoptosis, all of which are considered to be hallmarks of CKD. The list of all the miRNAs that were identified to be regulated in the kidney tissues of all the experimental mice groups analysed by Fireplex miRNA assay can be found in the Supplementary Data (Supplementary Tables S4, S5).

**TABLE 2:** Panther gene ontology pathway analyses.

**Pathways predicted by PANTHER online meta-analyses**	**No of genes involved**
Wnt signalling pathway (P00057)	138
Inflammation mediated by chemokine and cytokine signalling pathway (P00031)	94
Angiogenesis (P00005)	91
Cadherin signalling pathway (P00012)	77
Integrin signalling pathway (P00034)	73
PDGF signalling pathway (P00047)	71
EGF receptor signalling pathway (P00018)	69
FGF signalling pathway (P00021)	58
TGF-beta signalling pathway (P00052)	53
Apoptosis signalling pathway (P00006)	52
p53 pathway (P00059)	44
Interleukin signalling pathway (P00036)	42
Endothelin signalling pathway (P00019)	40
T cell activation (P00053)	39
Ras Pathway (P04393)	35
B cell activation (P00010)	33
Oxidative stress response (P00046)	31
VEGF signalling pathway (P00056)	29
Toll receptor signalling pathway (P00054)	26
PI3 kinase pathway (P00048)	24
Notch signalling pathway (P00045)	24
Interferon-gamma signalling pathway (P00035)	23
FAS signalling pathway (P00020)	15
Hypoxia response via HIF activation (P00030)	14
GABA-B receptor II signalling (P05731)	13
p53 pathway by glucose deprivation (P04397)	13
Histamine H1 receptor mediated signalling pathway (P04385)	13
Heterotrimeric G-protein signalling pathway-rod outer segment phototransduction (P00028)	11
Hedgehog signalling pathway (P00025)	11
General transcription regulation (P00023)	11
JAK/STAT signalling pathway (P00038)	10
Beta3 adrenergic receptor signalling pathway (P04379)	10
Glycolysis (P00024)	10
DNA replication (P00017)	9
Angiotensin II-stimulated signalling through G proteins and beta-arrestin (P05911)	8
Cell cycle (P00013)	7
P53 pathway feedback loops 1 (P04392)	6

*Predicted pathways regulated by miRNAs downregulated by MSC-EVs in AA mice. The top 37 pathways have been selected on the basis of number of genes involved in kidney injury.*

**TABLE 3:** Panther pathway enrichment analyses on validated target genes of differentially expressed miRNAs in AA mice treated with MSC-EVs.

**Pathways validated by PANTHER online meta-analyses**	**No of genes involved**
Angiogenesis (P00005)	12
Apoptosis signalling pathway (P00006)	10
Integrin signalling pathway (P00034)	10
PDGF signalling pathway (P00047)	9
CCKR signalling map (P06959)	9
TGF-beta signalling pathway (P00052)	8
Gonadotropin-releasing hormone receptor pathway (P06664)	8
p53 pathway (P00059)	7
p53 pathway feedback loops 2 (P04398)	7
Inflammation mediated by chemokine and cytokine signalling pathway (P00031)	6
FGF signalling pathway (P00021)	6
FAS signalling pathway (P00020)	6
EGF receptor signalling pathway (P00018)	6
Oxidative stress response (P00046)	6

## Discussion

The current study demonstrates the capacity of MSC-EVs to inhibit the progression of kidney injury in a CKD murine model of AAN. Following MSC-EV treatment, mice showed an overall improvement in kidney function, as reflected by a significant reduction of blood creatinine, and BUN levels. In addition, MSC-EVs significantly reduced tubular necrosis, interstitial fibrosis and favoured renal regeneration. Furthermore, a marked reduction in inflammatory cells, active fibroblasts, and pericytes was also observed after MSC-EV treatment.

Over the last decade, the role of stem-cell derived EVs, as an alternative strategy for tissue regeneration, has evolved tremendously. In particular, MSC-EVs have been extensively studied in various experimental disease models (Grange et al., 2019a). For instance, in the classical CKD model of ureter urinary obstruction (UUO), intravenous administration of MSC-EVs not only improved renal function in a span of two weeks, but also alleviated tubular injury and interstitial fibrosis (He et al., 2015). Interestingly, in a porcine model of renovascular disease (RVD), a more diverse model of CKD, a single dose of MSC-EVs alleviated renal inflammation through the regulation of pro and anti-inflammatory cytokines, contributing to recovery (Eirin et al., 2017).

Alternative sources of MSC-EVs have also proven to be effective. For example, urine derived MSC-EVs reduced podocyte and tubular epithelial cell apoptosis and increased proliferation of glomerular endothelial cells, therefore exhibiting an overall regenerative effect in a rat model of diabetic nephropathy (Jiang et al., 2016). Along the same line, Nagaishi et al. (2016) showed that administration of MSC exosomes under the renal capsule, in a similar mouse model of diabetic nephropathy, resulted in equivalent anti-apoptotic effects and general improvement of renal morphology.

More recently, our group reported the therapeutic effects of multiple sources of stem-cell derived EVs, in a mouse model of streptozotocin induced diabetic nephropathy (Grange et al., 2019b). Grange et al. (2019b) showed that the administration of EVs derived from both MSCs and HLSCs in diabetic mice, significantly reduced, to near normal levels, the elevated kidney functional parameters plasma creatinine, BUN and albumin/creatinine excretion. In addition, the authors also observed a significant reduction of fibrosis both at a protein and molecular level, as a result of MSC-EV and HLSC-EV treatment. A comparative miRNA expression analysis between both EV types highlighted the presence of some common and some uncommon miRNAs linked to the regulation of pro-fibrotic genes. These findings further supported the anti-fibrotic effect of MSC-EV, in a curative model of CKD (Grange et al., 2019b). In addition, the regenerative effect of HLSC-EVs was also reported by our lab, in an alternative model of CKD. We demonstrated that in the CKD model of AAN, HLSC-EVs not only improved the overall kidney function, but also alleviated interstitial fibrosis and contributed to tubular repair. The underlying repairing mechanisms were partially attributed to the regulation of genes and miRNAs involved in the progression of the disease (Kholia et al., 2018).

In the current study, using the same CKD model of AAN, we attempted to evaluate the effect of MSC-EVs. We particularly sought to identify similarities in the amelioration of kidney damage and possible differences in the underlying molecular mechanisms between MSC and HLSC EV sources.

The pathogenesis of CKD primarily involves dysfunction of the local parenchyma in the form of tubular damage that eventually progresses to necrosis and formation of hyaline casts (Grange et al., 2019a). This phase of kidney injury is directly correlated with an impairment of renal function, observed through elevated blood BUN and creatinine levels as well as loss of body weight as observed in various experimental models of AAN (Zhou et al., 2010; Yuan et al., 2011; Bruno et al., 2012; Huang et al., 2013; Grange et al., 2019b).

In the current study, the administration of AA increased tubular damage and necrosis, as well as the levels of blood creatinine and BUN. A gradual loss of mouse body weight, over the course of the time, was also observed. Treatment with MSC-EVs ameliorated tubular necrosis and reduced blood BUN and creatinine levels. This effect was similar to the one previously described following HLSC-EV treatment (Kholia et al., 2018). However, at variance with HLSC-EVs, no significant improvement in body weight was observed in mice treated with MSC-EVs.

The activation of inflammatory, fibrotic, and reparative processes occur simultaneously to promote regeneration and repair, following an insult. An increase in the infiltration of inflammatory and pro-fibrotic cells can impair tissue regeneration and lead to fibrosis (Black et al., 2019). Renal fibrosis characterised by progressive tissue scarring is a hallmark of CKD and a prominent feature of AAN. Most of the patient cases of AAN, described in the literature, link chronic kidney failure with fibrosis accompanied with interstitial inflammatory infiltrates (Lo et al., 2004; Yang et al., 2007, 2012). In addition, various experimental models of AAN also describe fibrosis, as a common feature initiated mainly by the combination of inflammatory cells and infiltration/activation of resident fibroblasts (Pozdzik et al., 2008a; Sato and Yanagita, 2017).

Pozdzik et al. (2008a) reported the accumulation of cells positive for vimentin and α-smooth muscle actin, both markers of activated fibroblasts, in the renal interstitium of rats intoxicated with AA. This was further confirmed by Huang et al. (2013) in their mouse model of AAN, whereby an influx of FSP-1 positive cells was observed in the kidneys of AA treated mice. Furthermore, infiltration/activation of fibroblasts was accompanied by the inflow of CD45 positive inflammatory cells, as well as activated mononuclear and cytotoxic T cells (Pozdzik et al., 2008a; Huang et al., 2013).

Along the same line, in our model of AAN, intoxication with AA induced an influx of inflammatory cells (CD45 positive cells) and myofibroblast activation (FSP-1 positive cells, and α-SMA staining) in the renal interstitium, which was alleviated by HLSC-EV treatment (Kholia et al., 2018). Interestingly, the same effect was observed in our current study with MSC-EVs. Mice that received treatment with MSC-EVs, showed a significantly lower expression of CD45 positive cells, FSP-1 and α-SMA positive myofibroblasts. As NSG mice lack an adaptive immune system due to their genetic background (Shultz et al., 2005), we speculate that, the CD45 positive cells are likely to be part of the innate immune system. As a severe kidney injury was observed in NSG mice following AA injection, we speculate that the mechanism of injury could be mainly related to the innate immune system. To explore the role of the adaptive immune system, immunocompetent mice need to be used. However, in the current study, as the MSC-EVs were derived from human origin, immunodeficient mice had to be used to avoid an immune reaction related to xenogenic material. Overall, our data indicate that MSC-EVs attenuate the innate immune response ameliorating AA induced kidney injury.

Another cell type known to play an important role in fibrosis is the pericyte. They have been identified as a major source of myofibroblasts in interstitial fibrosis (Wang et al., 2017). These perivascular cells rich in PDGFRβ mainly provide support to the vasculature under physiological conditions. However, during a pathological insult in the kidney, they tend to migrate toward the renal interstitium and undergo transition into myofibroblasts positive for α-SMA (Wang et al., 2017). In the current model, mice intoxicated with AA had a significantly high number of pericyte/myofibroblast transitioned cells (positive for PDGFRβ/α-SMA), which was significantly reduced following MSC-EV treatment. The reduction in interstitial fibrosis was also observed at a molecular level, whereby a downregulation of the pro-fibrotic genes α*-Sma*, *Col1a1*, and *Tgfb1* was detected in kidneys of mice treated with MSC-EVs. *In vitro* experiments on fibroblasts co-cultured with AA injured mTECs further supported a role of MSC-EVs as mediators of myofibroblast activation, as downregulation of pro-fibrotic genes was observed following MSC-EV treatment. Furthermore, a marked increase in proliferating cells, as evaluated by PCNA staining and a significant reduction in apoptotic cells evaluated by TUNEL staining, was observed in mice treated with MSC-EVs, further supporting the activation of a renal pro-regenerative programme. These findings demonstrate the anti-inflammatory/anti-fibrotic properties of MSC-EVs, previously described for HLSC-EVs in various models of CKD (Kholia et al., 2018; Grange et al., 2019b).

TGFβ-1, a key factor in fibrosis was subsequently analysed in our model. It is a major regulator of the pro-fibrotic process leading to the development of scar tissue and ultimately end stage renal disease (Jadot et al., 2017). The dysregulation of TGFβ-1 has been reported in various experimental models of AAN (Pozdzik et al., 2008b; Wang et al., 2008). In line with these studies, we also observed a significant upregulation of TGFβ-1, at molecular level, in mice damaged with AA. Interestingly, this upregulation was further reverted following treatment with MSC-EVs. A similar trend was also observed in the expression of the *ltbp1* gene which encodes for the LTBP1 protein, involved in the activation of TGFβ-1. Of note, these data suggest a possible mechanism of action of MSC-EVs in the regulation of TGFβ-1, which itself is a key stone in the progression of inflammation and fibrosis (Wang et al., 2008; Black et al., 2019).

miRNAs are non-coding RNA molecules, that function as post-transcriptional regulators, blocking target mRNAs. Their role in regeneration has been gaining an increasing interest over the past decade (Ignarski et al., 2019). Various studies have reported the altered expression of miRNAs in renal tissue during the progression of acute and CKDs, both in human and animal models (Trionfini et al., 2015). In addition, miRNAs enriched in EVs, have been shown to exert their biological action by being directly transferred into recipient cells or indirectly by influencing the cells genetic content (Ratajczak et al., 2006; Deregibus et al., 2007). We therefore, sought to investigate, the dysregulation of miRNAs in our current model of AAN. Seven miRNAs were identified to be upregulated in AA intoxicated kidneys, and downregulated following MSC-EV treatment. Interestingly, these miRNAs have been reported to play a role in kidney fibrosis. For instance, Thum et al. (2008) first reported the overexpression of miR21 in a fibrosis model of heart failure, while subsequent studies demonstrated its involvement in various other models, including kidney fibrosis (Liu et al., 2010; Denby et al., 2011). In another study, the upregulation of miR34a was found to be elevated in the interstitium of mouse fibrotic kidneys subjected to UUO injury (Zhou et al., 2014). In addition, both miR132-3p and miR214 have been found to be upregulated in various models of kidney diseases and associated with inflammation, apoptosis, myofibroblast activation, and dysregulation of the extracellular matrix (Denby et al., 2011, 2014; Bijkerk et al., 2016). Furthermore, attenuation of these miRNAs through inhibition or silencing has been shown to ameliorate the various forms of kidney diseases by downregulating pathways and processes favoring the progression of injury and fibrosis (Denby et al., 2011; Liu et al., 2019).

Bioinformatic meta-analysis was performed on the predicted and validated target genes of these 7 miRNAs for the relative pathways to be identified. Through online Panther pathway analysis, we identified over 30 predicted pathways out of which some were linked with kidney injury and paralleled with the ones regulated by HLSC-EVs in our previous study (Kholia et al., 2018). Among some of these pathways, the WNT/beta-catenin signalling pathway, chemokine/cytokine pathway, and the TGFβ signalling pathway were identified. On the other hand, the validated pathways regulated by the 7 miRNAs mentioned above, were much less (14 pathways) in comparison with the validated pathways regulated by HLSC-EVs (data not shown). Nonetheless, this study allowed us to identify validated pathways that were specifically regulated by MSC-EVs which include: apoptosis, TGFβ, FAS, and P53 signalling pathways amongst others that have been reported to play a role in CKD (Chung and Lan, 2015; Kholia et al., 2018; Black et al., 2019; Grange et al., 2019b). Moreover, the comparison between miRNAs regulated by MSC-EVs in the current study and HLSC-EVs in our previous study (Kholia et al., 2018), showed no overlap, suggesting that the molecular targets of stem cell derived EVs could differ despite their overall common therapeutic effect.

Various studies have been performed in our lab to identify the active biological molecules responsible for the therapeutic effects observed. For instance, proteomics studies, performed by Collino et al. (2017) on MSC-EV fractions, revealed the presence of cytokines, chemokine receptors such as CXCR1, CXCR6 etc; interleukins such as IL13, IL10, IL 4, that are involved in regulating multiple immunomodulatory, and anti-inflammatory pathways. In an earlier study by Collino et al. (2015), EVs from wild-type MSCs induced morphological and functional recovery of the kidneys in a mouse model of AKI, whereas EVs from drosha-knockdown MSCs that had a global downregulation of microRNAs were ineffective. They showed, through RNA sequencing and gene ontology analysis that kidney genes dysregulated post AKI (that mainly influenced pathways related to inflammation, matrix-receptor interaction, among others) were reverted after treatment with wild-type MSC-EVs but not EVs from drosha-knockdown MSCs. Therefore, confirming the contribution of miRNAs towards MSC-EV biological activity. In a recent study by Grange et al. (2019b), molecular analysis of MSC-EVs revealed enrichment with miRNAs that regulated various inflammatory and pro-fibrotic pathways therefore further confirming part of the EV therapeutic effects to the miRNA content. Although, multiple reports have been published dissecting the contents of stem cell derived EVs to identify the active content responsible for their biological activity, the demonstration that single or multiple component are responsible for their specific function are still very challenging.

## Conclusion

Our data suggest that multiple injections of MSC-EVs not only improved renal function, but also ameliorated kidney fibrosis, and tubular necrosis, possibly by attenuating the infiltration of inflammatory cells and myofibroblasts, as well as by reducing the transition of pericytes to myofibroblasts. Although the various regenerative effects of MSC-EVs on AA injured mice were similar to those previously observed with HLSC-EVs on the same model of AAN (Kholia et al., 2018), the biological effect exerted by both EV sources, can be ascribed to different underlying molecular mechanisms.

## Data Availability Statement

The raw data supporting the conclusions of this article will be made available by the authors, without undue reservation, to any qualified researcher.

## Ethics Statement

Animal studies were conducted in accordance with the National Institute of Health Guidelines for the Care and Use of Laboratory Animals. All procedures were approved by the Ethics Committee of the University of Turin and the Italian Health Ministry (authorisation number: 766/2016-PR).

## Author Contributions

SK, MH, and GC contributed conception and design of the study, acquisition, analyses and interpretation of data, as well as drafting the manuscript. SB, MC, EP, MT, FA, and MD contributed toward acquisition and analyses of data. MB and PQ contributed toward interpretation of data, manuscript preparation, and final approval. All authors contributed toward the manuscript revision, as well as reading and approving the submitted version.

## Conflict of Interest

GC is member of the Scientific Advisory Board of Unicyte AG. MH, MD, and GC are named inventors in related patents. The remaining authors declare that the research was conducted in the absence of any commercial or financial relationships that could be construed as a potential conflict of interest.

## Abbreviations

α -Sma: alpha smooth muscle actin
AA: aristolochic acid
AAN: aristolochic acid nephropathy
AKI: acute kidney injury
BEN: Balkan endemic nephropathy
BUN: Blood urea nitrogen
CHN: Chinese herbal nephropathy
CKD: chronic kidney disease
DMEM: Dulbecco’s minimum essential medium
DMSO: dimethyl sulfoxide
EVs: extracellular vesicles
HLSC: human liver stem cell
LTBP1: latent TGFb binding protein 1
MFI: mean fluorescence intensity
mkCF: mouse kidney cortical fibroblast
MSC: mesenchymal stem cells
mTEC: mouse tubular epithelial cell
PDGF: platelet derived growth factor
TGFb: transforming growth factor beta
UUO: Unilateral ureter injury
WNT: Wingless-related integration site.
