# Supplementary material for: Mesenchymal Stem Cell Derived Extracellular Vesicles Ameliorate Kidney Injury in Aristolochic Acid Nephropathy
Source: Front Cell Dev Biol. 2020 Mar 24;8:188. doi: 10.3389/fcell.2020.00188 (PMC7105599; doi:10.3389/fcell.2020.00188)
Supplement: Supplementary file 1 [file Table_1.DOCX]

Supplementary Material

**Mesenchymal stem cell derived extracellular vesicles ameliorate kidney injury in aristolochic acid nephropathy**

Sharad Kholia^1,3#^, Maria Beatriz Herrera Sanchez^2,3#^, Massimo Cedrino^2,3^, Elli Papadimitrou^3^, Marta Tapparo^1,3^, Maria Chiara Deregibus^2,3^, Stefania Bruno^1^, Federica Antico^4^, Maria Felice Brizzi^1^, Peter J Quesenberry^5^ and Giovanni Camussi^1,2*^

**
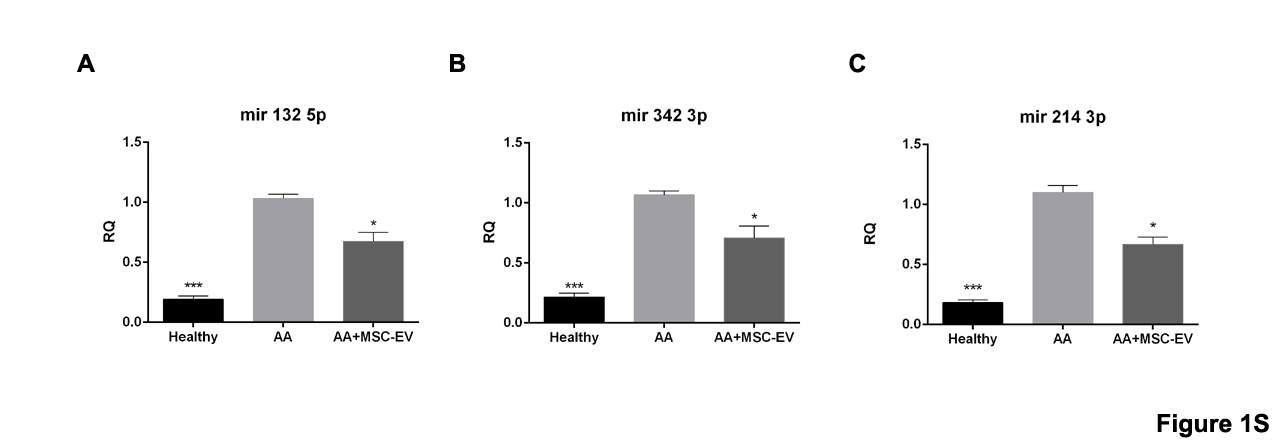
**

**Supplementary Figure 1:** **Validation of miRNAs identified through the Fireplex miRNA assay that were regulated by MSC-EVs in AA treated mice kidneys.** Out of the 7 miRNAs identified to be regulated by MSC-EVs in AA treated mice kidneys we validated three miRNAs by RT-PCR. Briefly, Total RNA was isolated from ten 8 µm formalin fixed parafilm embedded (FFPE) sections (n=3/condition) using the RecoverALL^TM^ Total Nucleic acid isolation kit for FFPE according to the manufacturer’s protocol. The total RNA from each mouse (200 ng of input RNA) was reverse transcribed and the cDNA subjected to RT-PCR for validation of specific miRNAs. ***p< 0.001 control vs AA, and *p<0.05 AA+MSC-EVs vs AA. Data are expressed as mean ± SEM; a one way ANOVA with Bonferroni’s multi comparison test was performed.

**Supplementary Table S1.** Differentially expressed miRNAs, screened by FirePlex miRNA assay, in AA mice with respect to healthy control mice.

|  | **MFI** | |  | **Bonferroni’s** |  |
| --- | --- | --- | --- | --- | --- |
| **probe** | **AA** | **Control** | **fold** | **adj p** | **raw p** |
| hsa-mir-132-3p | 560.854 | 171.142 | 3.277127 | 8.28E-06 | 1.27E-07 |
| hsa-mir-34c-5p | 526.474 | 96.947 | 5.430534 | 1.77E-05 | 2.72E-07 |
| hsa-mir-214-3p | 1663.838 | 528.558 | 3.147882 | 2.11E-05 | 3.25E-07 |
| mmu-mir-212-3p | 71.954 | 19.399 | 3.70916 | 0.000376 | 5.79E-06 |
| hsa-mir-342-3p | 180.645 | 54.591 | 3.309062 | 0.000506 | 7.79E-06 |
| hsa-mir-486-5p | 588.008 | 95.229 | 6.174674 | 0.000566 | 8.72E-06 |
| hsa-mir-21-5p | 597.266 | 188.058 | 3.175967 | 0.00161 | 2.48E-05 |
| hsa-mir-34a-5p | 3751.095 | 2232.668 | 1.680095 | 0.00221 | 3.41E-05 |
| hsa-mir-192-5p | 3174.123 | 1597.979 | 1.986336 | 0.00257 | 3.96E-05 |
| hsa-mir-194-5p | 3767.312 | 2440.495 | 1.543667 | 0.00367 | 5.66E-05 |
| mmu-mir-489-3p | 56.17 | 22.534 | 2.492678 | 0.00623 | 9.62E-05 |
| hsa-mir-206 | 13.831 | 2.541 | 5.443133 | 0.016 | 0.000249 |
| mmu-mir-378a-3p | 975.537 | 1475.58 | 0.661121 | 0.0228 | 0.000354 |

**Supplementary Table S2**. Differentially expressed miRNAs, screened by FirePlex miRNA assay, in

AA mice in respect to AA+MSC-EVs mice. miRNAs that is restored as healthy control level are highlighted in bold.

|  | **MFI** | |  | **Bonferroni’s** |  |
| --- | --- | --- | --- | --- | --- |
| **probe** | **AA** | **AA+MSC-EVs** | **fold** | **adj p** | **raw p** |
| hsa-mir-17-5p | 3285.614 | 1701.496 | 0.518 | 1.08E-09 | 1.66E-11 |
| hsa-mir-93-5p | 3798.27 | 2043.892 | 0.538 | 1.84E-07 | 2.84E-09 |
| hsa-mir-194-5p | 2440.495 | 1194.146 | 0.489 | 2.41E-07 | 3.71E-09 |
| hsa-mir-23b-3p | 3364.327 | 1611.314 | 0.479 | 3.69E-07 | 5.68E-09 |
| hsa-mir-27a-3p | 1823.84 | 816.716 | 0.448 | 2.32E-06 | 3.57E-08 |
| hsa-mir-143-3p | 4676.542 | 2226.439 | 0.476 | 6.71E-06 | 1.03E-07 |
| hsa-mir-130a-3p | 4507.384 | 2069.915 | 0.459 | 2.59E-05 | 3.99E-07 |
| hsa-mir-191-5p | 5457.784 | 2868.554 | 0.526 | 5.22E-05 | 8.04E-07 |
| **hsa-mir-21-5p** | 597.266 | 256.129 | 0.429 | 6.69E-05 | 1.03E-06 |
| hsa-mir-18a-5p | 147.931 | 69.229 | 0.468 | 6.81E-05 | 1.05E-06 |
| hsa-mir-30a-5p | 795.751 | 330.304 | 0.415 | 9.10E-05 | 1.40E-06 |
| **hsa-mir-34a-5p** | 3751.095 | 2105.692 | 0.561 | 9.58E-05 | 1.47E-06 |
| **hsa-mir-34c-5p** | 526.474 | 220.901 | 0.42 | 0.000148 | 2.27E-06 |
| hsa-mir-146a-5p | 952.814 | 381.162 | 0.4 | 0.000277 | 4.27E-06 |
| hsa-mir-145-5p | 3208.355 | 1551.015 | 0.483 | 0.000337 | 5.19E-06 |
| hsa-mir-125b-5p | 47.156 | 22.335 | 0.474 | 0.000384 | 5.91E-06 |
| hsa-let-7e-5p | 1522.518 | 712.839 | 0.468 | 0.000411 | 6.32E-06 |
| hsa-let-7g-5p | 530.942 | 267.366 | 0.504 | 0.000453 | 6.96E-06 |
| hsa-mir-126-3p | 2275.168 | 1095.956 | 0.482 | 0.000453 | 6.97E-06 |
| hsa-mir-15b-5p | 549.666 | 243.937 | 0.444 | 0.000498 | 7.67E-06 |
| hsa-mir-200c-3p | 1113.427 | 496.996 | 0.446 | 0.00063 | 9.69E-06 |
| hsa-mir-200a-3p | 2852.188 | 1489.045 | 0.522 | 0.00182 | 2.81E-05 |
| **hsa-mir-132-3p** | 560.854 | 243.549 | 0.434 | 0.002 | 3.09E-05 |
| hsa-mir-192-5p | 1597.979 | 718.889 | 0.45 | 0.00274 | 4.22E-05 |
| **hsa-mir-342-3p** | 180.645 | 71.094 | 0.394 | 0.00286 | 4.40E-05 |
| hsa-mir-100-5p | 418.132 | 167.777 | 0.401 | 0.00296 | 4.55E-05 |
| hsa-mir-15a-5p | 1487.638 | 666.56 | 0.448 | 0.00353 | 5.44E-05 |
| hsa-mir-484 | 1432.872 | 702.332 | 0.49 | 0.00551 | 8.50E-05 |
| hsa-mir-10a-5p | 565.572 | 250.754 | 0.443 | 0.00623 | 9.61E-05 |
| hsa-mir-98-5p | 177.727 | 96.621 | 0.544 | 0.0069 | 0.000106 |
| hsa-mir-29a-3p | 2811.275 | 1399.209 | 0.498 | 0.00906 | 0.00014 |
| hsa-mir-181a-5p | 2395.39 | 1061.544 | 0.443 | 0.00945 | 0.000146 |
| mmu-mir-378a-3p | 975.537 | 439.332 | 0.45 | 0.00993 | 0.000153 |
| **mmu-mir-212-3p** | 71.954 | 34.332 | 0.477 | 0.0122 | 0.000188 |
| hsa-let-7c-5p | 7585.544 | 4475.797 | 0.59 | 0.0126 | 0.000195 |
| **hsa-mir-214-3p** | 1663.838 | 644.486 | 0.387 | 0.0409 | 0.000642 |

**Supplementary Table S3**. **Panther gene ontology pathway analyses.** Predicted pathways regulated by miRNAs downregulated by MSC-EVs in AA mice**.**

|  |  |
| --- | --- |
| Wnt signaling pathway (P00057) | 138 |
| Gonadotropin-releasing hormone receptor pathway (P06664) | 102 |
| Inflammation mediated by chemokine and cytokine signaling pathway (P00031) | 94 |
| Angiogenesis (P00005) | 91 |
| CCKR signaling map (P06959) | 82 |
| Cadherin signaling pathway (P00012) | 77 |
| Integrin signalling pathway (P00034) | 73 |
| PDGF signaling pathway (P00047) | 71 |
| Heterotrimeric G-protein signaling pathway-Gi alpha and Gs alpha mediated pathway (P00026) | 70 |
| EGF receptor signaling pathway (P00018) | 69 |
| Alzheimer disease-presenilin pathway (P00004) | 68 |
| FGF signaling pathway (P00021) | 58 |
| Huntington disease (P00029) | 55 |
| TGF-beta signaling pathway (P00052) | 53 |
| Heterotrimeric G-protein signaling pathway-Gq alpha and Go alpha mediated pathway (P00027) | 53 |
| Apoptosis signaling pathway (P00006) | 52 |
| p53 pathway (P00059) | 44 |
| Parkinson disease (P00049) | 44 |
| Interleukin signaling pathway (P00036) | 42 |
| Nicotinic acetylcholine receptor signaling pathway (P00044) | 40 |
| Endothelin signaling pathway (P00019) | 40 |
| T cell activation (P00053) | 39 |
| Alzheimer disease-amyloid secretase pathway (P00003) | 35 |
| Ras Pathway (P04393) | 35 |
| B cell activation (P00010) | 33 |
| Metabotropic glutamate receptor group III pathway (P00039) | 32 |
| p53 pathway feedback loops 2 (P04398) | 31 |
| Oxidative stress response (P00046) | 31 |
| Cytoskeletal regulation by Rho GTPase (P00016) | 30 |
| VEGF signaling pathway (P00056) | 29 |
| Ionotropic glutamate receptor pathway (P00037) | 27 |
| Ubiquitin proteasome pathway (P00060) | 26 |
| Toll receptor signaling pathway (P00054) | 26 |
| Muscarinic acetylcholine receptor 2 and 4 signaling pathway (P00043) | 25 |
| Muscarinic acetylcholine receptor 1 and 3 signaling pathway (P00042) | 25 |
| Thyrotropin-releasing hormone receptor signaling pathway (P04394) | 24 |
| PI3 kinase pathway (P00048) | 24 |
| Notch signaling pathway (P00045) | 24 |
| 5HT2 type receptor mediated signaling pathway (P04374) | 24 |
| Axon guidance mediated by netrin (P00009) | 24 |
| Interferon-gamma signaling pathway (P00035) | 23 |
| Blood coagulation (P00011) | 23 |
| p38 MAPK pathway (P05918) | 22 |
| Insulin/IGF pathway-protein kinase B signaling cascade (P00033) | 22 |
| Synaptic vesicle trafficking (P05734) | 20 |
| Dopamine receptor mediated signaling pathway (P05912) | 20 |
| Oxytocin receptor mediated signaling pathway (P04391) | 19 |
| Metabotropic glutamate receptor group II pathway (P00040) | 18 |
| 5HT1 type receptor mediated signaling pathway (P04373) | 18 |
| Transcription regulation by bZIP transcription factor (P00055) | 17 |
| Adrenaline and noradrenaline biosynthesis (P00001) | 16 |
| Opioid proopiomelanocortin pathway (P05917) | 15 |
| Beta1 adrenergic receptor signaling pathway (P04377) | 15 |
| FAS signaling pathway (P00020) | 15 |
| Nicotine pharmacodynamics pathway (P06587) | 14 |
| Opioid proenkephalin pathway (P05915) | 14 |
| Beta2 adrenergic receptor signaling pathway (P04378) | 14 |
| 5HT4 type receptor mediated signaling pathway (P04376) | 14 |
| Hypoxia response via HIF activation (P00030) | 14 |
| GABA-B receptor II signaling (P05731) | 13 |
| Opioid prodynorphin pathway (P05916) | 13 |
| p53 pathway by glucose deprivation (P04397) | 13 |
| Histamine H1 receptor mediated signaling pathway (P04385) | 13 |
| Insulin/IGF pathway-mitogen activated protein kinase kinase/MAP kinase cascade (P00032) | 13 |
| Alpha adrenergic receptor signaling pathway (P00002) | 12 |
| Enkephalin release (P05913) | 12 |
| Metabotropic glutamate receptor group I pathway (P00041) | 12 |
| Axon guidance mediated by Slit/Robo (P00008) | 12 |
| Plasminogen activating cascade (P00050) | 11 |
| Heterotrimeric G-protein signaling pathway-rod outer segment phototransduction (P00028) | 11 |
| Hedgehog signaling pathway (P00025) | 11 |
| General transcription regulation (P00023) | 11 |
| Cortocotropin releasing factor receptor signaling pathway (P04380) | 10 |
| JAK/STAT signaling pathway (P00038) | 10 |
| Beta3 adrenergic receptor signaling pathway (P04379) | 10 |
| Glycolysis (P00024) | 10 |
| Endogenous cannabinoid signaling (P05730) | 9 |
| De novo purine biosynthesis (P02738) | 9 |
| 5HT3 type receptor mediated signaling pathway (P04375) | 9 |
| DNA replication (P00017) | 9 |
| Angiotensin II-stimulated signaling through G proteins and beta-arrestin (P05911) | 8 |
| Histamine H2 receptor mediated signaling pathway (P04386) | 8 |
| 5-Hydroxytryptamine degredation (P04372) | 8 |
| Pyruvate metabolism (P02772) | 7 |
| Cell cycle (P00013) | 7 |
| Vitamin D metabolism and pathway (P04396) | 6 |
| De novo pyrimidine deoxyribonucleotide biosynthesis (P02739) | 6 |
| P53 pathway feedback loops 1 (P04392) | 6 |
| Circadian clock system (P00015) | 6 |
| Axon guidance mediated by semaphorins (P00007) | 6 |
| Salvage pyrimidine ribonucleotides (P02775) | 5 |
| N-acetylglucosamine metabolism (P02756) | 5 |
| Heme biosynthesis (P02746) | 5 |
| De novo pyrimidine ribonucleotides biosythesis (P02740) | 5 |
| TCA cycle (P00051) | 5 |
| Coenzyme A biosynthesis (P02736) | 5 |
| Androgen/estrogene/progesterone biosynthesis (P02727) | 5 |
| General transcription by RNA polymerase I (P00022) | 5 |
| Cholesterol biosynthesis (P00014) | 5 |

**Supplementary Table S4**. Data showing all the differentially expressed miRNAs, screened by FirePlex miRNA assay, in AA mice with respect to healthy control mice.

| probe | fold | adj p | raw p | **AA** | **Control** | fold-spread | inter-group CV |
| --- | --- | --- | --- | --- | --- | --- | --- |
| hsa-mir-132-3p | 0.305 | 8.28E-06 | 1.27E-07 | 560.854 | 171.142 | 1.153 | 0.532 |
| hsa-mir-34c-5p | 0.184 | 1.77E-05 | 2.72E-07 | 526.474 | 96.947 | 1.247 | 0.689 |
| hsa-mir-214-3p | 0.318 | 2.11E-05 | 3.25E-07 | 1663.838 | 528.558 | 1.16 | 0.518 |
| mmu-mir-212-3p | 0.27 | 0.000376 | 5.79E-06 | 71.954 | 19.399 | 1.256 | 0.575 |
| hsa-mir-342-3p | 0.302 | 0.000506 | 7.79E-06 | 180.645 | 54.591 | 1.252 | 0.536 |
| hsa-mir-486-5p | 6.175 | 0.000566 | 8.72E-06 | 95.229 | 588.008 | 1.369 | 0.721 |
| hsa-mir-21-5p | 0.315 | 0.00161 | 2.48E-05 | 597.266 | 188.058 | 1.268 | 0.521 |
| hsa-mir-34a-5p | 0.595 | 0.00221 | 3.41E-05 | 3751.095 | 2232.668 | 1.123 | 0.254 |
| hsa-mir-192-5p | 1.986 | 0.00257 | 3.96E-05 | 1597.979 | 3174.123 | 1.169 | 0.33 |
| hsa-mir-194-5p | 1.544 | 0.00367 | 5.66E-05 | 2440.495 | 3767.312 | 1.108 | 0.214 |
| mmu-mir-489-3p | 0.401 | 0.00623 | 9.62E-05 | 56.17 | 22.534 | 1.259 | 0.427 |
| hsa-mir-206 | 0.184 | 0.016 | 0.000249 | 13.831 | 2.541 | 1.582 | 0.69 |
| mmu-mir-378a-3p | 1.513 | 0.0228 | 0.000354 | 975.537 | 1475.58 | 1.119 | 0.204 |
| hsa-mir-455-5p | 2.19 | 0.0589 | 0.000933 | 45.652 | 99.959 | 1.305 | 0.373 |
| hsa-mir-223-3p | 0.371 | 0.0659 | 0.00105 | 142.127 | 52.79 | 1.395 | 0.458 |
| hsa-mir-146a-5p | 0.553 | 0.102 | 0.00166 | 952.814 | 527.32 | 1.244 | 0.287 |
| hsa-mir-200c-3p | 0.763 | 0.317 | 0.00585 | 1113.427 | 849.492 | 1.129 | 0.134 |
| hsa-mir-203a-3p | 2.457 | 0.356 | 0.00674 | 67.522 | 165.882 | 1.504 | 0.421 |
| hsa-mir-181a-5p | 0.761 | 0.372 | 0.00713 | 2395.39 | 1823.993 | 1.133 | 0.135 |
| hsa-mir-27a-3p | 0.785 | 0.387 | 0.00751 | 1823.84 | 1431.705 | 1.12 | 0.12 |
| hsa-let-7g-5p | 1.583 | 0.419 | 0.00831 | 530.942 | 840.313 | 1.225 | 0.226 |
| hsa-mir-532-3p | 0.741 | 0.523 | 0.0113 | 460.037 | 341.005 | 1.165 | 0.149 |
| hsa-mir-126-3p | 1.267 | 0.693 | 0.018 | 2275.168 | 2883.262 | 1.141 | 0.118 |
| hsa-mir-210-3p | 0.645 | 0.705 | 0.0186 | 187.79 | 121.199 | 1.277 | 0.216 |
| hsa-mir-17-5p | 1.068 | 0.767 | 0.0221 | 3285.614 | 3507.875 | 1.039 | 0.0327 |
| hsa-mir-29a-3p | 1.307 | 0.874 | 0.0314 | 2811.275 | 3673.477 | 1.184 | 0.133 |
| hsa-mir-18a-5p | 0.703 | 0.89 | 0.0334 | 147.931 | 104.043 | 1.237 | 0.174 |
| rno-mir-183-5p | 0.731 | 0.916 | 0.0373 | 46.045 | 33.644 | 1.215 | 0.156 |
| hsa-mir-30c-5p | 1.178 | 0.978 | 0.0569 | 6121.157 | 7213.504 | 1.127 | 0.0819 |
| hsa-mir-186-5p | 1.667 | 0.992 | 0.071 | 9.437 | 15.732 | 1.479 | 0.25 |
| hsa-mir-26b-5p | 1.489 | 0.997 | 0.0854 | 27.729 | 41.286 | 1.344 | 0.196 |
| hsa-mir-146b-5p | 0.571 | 0.999 | 0.11 | 247.088 | 141.211 | 1.639 | 0.273 |
| hsa-mir-30e-3p | 1.418 | 1 | 0.148 | 42.138 | 59.768 | 1.415 | 0.173 |
| hsa-mir-20b-5p | 1.393 | 1 | 0.177 | 1042.554 | 1452.083 | 1.433 | 0.164 |
| hsa-let-7e-5p | 0.892 | 1 | 0.179 | 1522.518 | 1357.979 | 1.133 | 0.0571 |
| hsa-mir-130a-3p | 0.933 | 1 | 0.189 | 4507.384 | 4206.543 | 1.08 | 0.0345 |
| mmu-mir-155-5p | 0.632 | 1 | 0.193 | 29.817 | 18.856 | 1.551 | 0.225 |
| hsa-mir-93-5p | 0.954 | 1 | 0.199 | 3798.27 | 3622.289 | 1.051 | 0.0237 |
| hsa-mir-125b-5p | 0.873 | 1 | 0.206 | 47.156 | 41.169 | 1.17 | 0.0678 |
| hsa-mir-19a-3p | 1.746 | 1 | 0.221 | 6.781 | 11.842 | 1.939 | 0.272 |
| hsa-mir-100-5p | 0.836 | 1 | 0.238 | 418.132 | 349.479 | 1.252 | 0.0894 |
| hsa-mir-134-5p | 1.345 | 1 | 0.261 | 1.839 | 2.473 | 1.403 | 0.147 |
| hsa-mir-409-3p | 4.698 | 1 | 0.291 | 0.454 | 2.135 | 4.495 | 0.649 |
| hsa-mir-98-5p | 1.101 | 1 | 0.305 | 177.727 | 195.671 | 1.144 | 0.0481 |
| hsa-mir-122-5p | 0.428 | 1 | 0.343 | 17.751 | 41.488 | 3.726 | 0.401 |
| mmu-mir-182-5p | 0.872 | 1 | 0.357 | 61.069 | 53.281 | 1.25 | 0.0681 |
| hsa-mir-138-5p | 0.729 | 1 | 0.371 | 34.938 | 25.461 | 1.602 | 0.157 |
| hsa-mir-145-5p | 1.072 | 1 | 0.382 | 3208.355 | 3438.484 | 1.127 | 0.0346 |
| hsa-mir-191-5p | 0.946 | 1 | 0.389 | 5457.784 | 5161.655 | 1.102 | 0.0279 |
| hsa-mir-484 | 1.078 | 1 | 0.412 | 1432.872 | 1544.721 | 1.146 | 0.0376 |
| hsa-mir-140-3p | 1.119 | 1 | 0.468 | 136.959 | 153.261 | 1.257 | 0.0562 |
| hsa-mir-10a-5p | 0.908 | 1 | 0.482 | 565.572 | 513.675 | 1.231 | 0.0481 |
| hsa-mir-142-3p | 1.144 | 1 | 0.489 | 2.072 | 2.371 | 1.342 | 0.0673 |
| hsa-mir-23b-3p | 0.969 | 1 | 0.494 | 3364.327 | 3258.73 | 1.072 | 0.0159 |
| hsa-mir-494-3p | 1.17 | 1 | 0.616 | 3.734 | 4.368 | 1.541 | 0.0782 |
| hsa-mir-200a-3p | 1.033 | 1 | 0.645 | 2852.188 | 2945.061 | 1.113 | 0.016 |
| hsa-mir-30a-5p | 1.048 | 1 | 0.687 | 795.751 | 833.603 | 1.193 | 0.0232 |
| hsa-mir-143-3p | 1.032 | 1 | 0.696 | 4676.542 | 4827.419 | 1.13 | 0.0159 |
| hsa-mir-130b-3p | 1.147 | 1 | 0.716 | 589.924 | 676.374 | 1.773 | 0.0683 |
| hsa-mir-16-5p | 0.972 | 1 | 0.745 | 7589.76 | 7374.955 | 1.133 | 0.0144 |
| hsa-mir-15b-5p | 1.028 | 1 | 0.815 | 549.666 | 565.006 | 1.197 | 0.0138 |
| hsa-mir-15a-5p | 0.969 | 1 | 0.851 | 1487.638 | 1441.887 | 1.292 | 0.0156 |
| hsa-mir-218-5p | 1.048 | 1 | 0.902 | 4.394 | 4.603 | 1.785 | 0.0232 |
| hsa-mir-320a | 1.006 | 1 | 0.956 | 967.853 | 973.629 | 1.176 | 0.00297 |
| hsa-let-7c-5p | 1.003 | 1 | 0.977 | 7585.544 | 7608.449 | 1.164 | 0.00151 |

**Supplementary Table S5**. Data showing all the differentially expressed miRNAs, screened by FirePlex miRNA assay, in AA mice with respect to AA+MSC-EVs mice.

| probe | fold | adj p | raw p | **AA** | **AA+MSC-EVs** | fold-spread | inter-group CV |
| --- | --- | --- | --- | --- | --- | --- | --- |
| hsa-mir-17-5p | 0.518 | 1.08E-09 | 1.66E-11 | 3285.614 | 1701.496 | 1.052 | 0.318 |
| hsa-mir-93-5p | 0.538 | 1.84E-07 | 2.84E-09 | 3798.27 | 2043.892 | 1.079 | 0.3 |
| hsa-mir-194-5p | 0.489 | 2.41E-07 | 3.71E-09 | 2440.495 | 1194.146 | 1.095 | 0.343 |
| hsa-mir-23b-3p | 0.479 | 3.69E-07 | 5.68E-09 | 3364.327 | 1611.314 | 1.099 | 0.352 |
| hsa-mir-27a-3p | 0.448 | 2.32E-06 | 3.57E-08 | 1823.84 | 816.716 | 1.125 | 0.381 |
| hsa-mir-143-3p | 0.476 | 6.71E-06 | 1.03E-07 | 4676.542 | 2226.439 | 1.136 | 0.355 |
| hsa-mir-130a-3p | 0.459 | 2.59E-05 | 3.99E-07 | 4507.384 | 2069.915 | 1.132 | 0.371 |
| hsa-mir-191-5p | 0.526 | 5.22E-05 | 8.04E-07 | 5457.784 | 2868.554 | 1.126 | 0.311 |
| hsa-mir-21-5p | 0.429 | 6.69E-05 | 1.03E-06 | 597.266 | 256.129 | 1.188 | 0.4 |
| hsa-mir-18a-5p | 0.468 | 6.81E-05 | 1.05E-06 | 147.931 | 69.229 | 1.165 | 0.362 |
| hsa-mir-30a-5p | 0.415 | 9.10E-05 | 1.40E-06 | 795.751 | 330.304 | 1.206 | 0.413 |
| hsa-mir-34a-5p | 0.561 | 9.58E-05 | 1.47E-06 | 3751.095 | 2105.692 | 1.13 | 0.281 |
| hsa-mir-34c-5p | 0.42 | 0.000148 | 2.27E-06 | 526.474 | 220.901 | 1.214 | 0.409 |
| hsa-mir-146a-5p | 0.4 | 0.000277 | 4.27E-06 | 952.814 | 381.162 | 1.234 | 0.429 |
| hsa-mir-145-5p | 0.483 | 0.000337 | 5.19E-06 | 3208.355 | 1551.015 | 1.174 | 0.348 |
| hsa-mir-125b-5p | 0.474 | 0.000384 | 5.91E-06 | 47.156 | 22.335 | 1.198 | 0.357 |
| hsa-let-7e-5p | 0.468 | 0.000411 | 6.32E-06 | 1522.518 | 712.839 | 1.181 | 0.362 |
| hsa-let-7g-5p | 0.504 | 0.000453 | 6.96E-06 | 530.942 | 267.366 | 1.168 | 0.33 |
| hsa-mir-126-3p | 0.482 | 0.000453 | 6.97E-06 | 2275.168 | 1095.956 | 1.186 | 0.35 |
| hsa-mir-15b-5p | 0.444 | 0.000498 | 7.67E-06 | 549.666 | 243.937 | 1.222 | 0.385 |
| hsa-mir-200c-3p | 0.446 | 0.00063 | 9.69E-06 | 1113.427 | 496.996 | 1.208 | 0.383 |
| hsa-mir-200a-3p | 0.522 | 0.00182 | 2.81E-05 | 2852.188 | 1489.045 | 1.175 | 0.314 |
| hsa-mir-132-3p | 0.434 | 0.002 | 3.09E-05 | 560.854 | 243.549 | 1.228 | 0.394 |
| hsa-mir-192-5p | 0.45 | 0.00274 | 4.22E-05 | 1597.979 | 718.889 | 1.24 | 0.379 |
| hsa-mir-342-3p | 0.394 | 0.00286 | 4.40E-05 | 180.645 | 71.094 | 1.307 | 0.435 |
| hsa-mir-100-5p | 0.401 | 0.00296 | 4.55E-05 | 418.132 | 167.777 | 1.291 | 0.427 |
| hsa-mir-15a-5p | 0.448 | 0.00353 | 5.44E-05 | 1487.638 | 666.56 | 1.273 | 0.381 |
| hsa-mir-484 | 0.49 | 0.00551 | 8.50E-05 | 1432.872 | 702.332 | 1.211 | 0.342 |
| hsa-mir-10a-5p | 0.443 | 0.00623 | 9.61E-05 | 565.572 | 250.754 | 1.279 | 0.386 |
| hsa-mir-98-5p | 0.544 | 0.0069 | 0.000106 | 177.727 | 96.621 | 1.182 | 0.296 |
| hsa-mir-29a-3p | 0.498 | 0.00906 | 0.00014 | 2811.275 | 1399.209 | 1.246 | 0.335 |
| hsa-mir-181a-5p | 0.443 | 0.00945 | 0.000146 | 2395.39 | 1061.544 | 1.244 | 0.386 |
| mmu-mir-378a-3p | 0.45 | 0.00993 | 0.000153 | 975.537 | 439.332 | 1.273 | 0.379 |
| mmu-mir-212-3p | 0.477 | 0.0122 | 0.000188 | 71.954 | 34.332 | 1.258 | 0.354 |
| hsa-let-7c-5p | 0.59 | 0.0126 | 0.000195 | 7585.544 | 4475.797 | 1.202 | 0.258 |
| hsa-mir-214-3p | 0.387 | 0.0409 | 0.000642 | 1663.838 | 644.486 | 1.341 | 0.442 |
| hsa-mir-16-5p | 0.598 | 0.0679 | 0.00108 | 7589.76 | 4535.114 | 1.233 | 0.252 |
| hsa-mir-140-3p | 0.407 | 0.0929 | 0.0015 | 136.959 | 55.761 | 1.449 | 0.421 |
| hsa-mir-203a-3p | 0.359 | 0.144 | 0.00239 | 67.522 | 24.215 | 1.556 | 0.472 |
| hsa-mir-223-3p | 0.385 | 0.29 | 0.00525 | 142.127 | 54.719 | 1.541 | 0.444 |
| hsa-mir-30e-3p | 0.344 | 0.398 | 0.00777 | 42.138 | 14.516 | 1.668 | 0.488 |
| hsa-mir-146b-5p | 0.406 | 0.489 | 0.0103 | 247.088 | 100.213 | 1.65 | 0.423 |
| mmu-mir-155-5p | 0.158 | 0.51 | 0.0109 | 29.817 | 4.719 | 2.195 | 0.727 |
| mmu-mir-489-3p | 0.348 | 0.565 | 0.0127 | 56.17 | 19.527 | 1.697 | 0.484 |
| hsa-mir-186-5p | 0.385 | 0.645 | 0.0158 | 9.437 | 3.629 | 1.693 | 0.445 |
| hsa-mir-30c-5p | 0.621 | 0.675 | 0.0171 | 6121.157 | 3798.794 | 1.294 | 0.234 |
| hsa-mir-26b-5p | 0.317 | 0.719 | 0.0193 | 27.729 | 8.777 | 1.755 | 0.519 |
| mmu-mir-182-5p | 0.271 | 0.741 | 0.0206 | 61.069 | 16.566 | 1.946 | 0.573 |
| hsa-mir-320a | 0.261 | 0.791 | 0.0238 | 967.853 | 252.92 | 1.937 | 0.586 |
| hsa-mir-532-3p | 0.451 | 0.853 | 0.029 | 460.037 | 207.552 | 1.563 | 0.378 |
| hsa-mir-138-5p | 0.506 | 0.941 | 0.0427 | 34.938 | 17.673 | 1.719 | 0.328 |
| hsa-mir-210-3p | 0.258 | 0.945 | 0.0436 | 187.79 | 48.38 | 2.208 | 0.59 |
| hsa-mir-20b-5p | 0.551 | 0.959 | 0.0478 | 1042.554 | 574.677 | 1.587 | 0.289 |
| hsa-mir-206 | 0.0352 | 0.962 | 0.049 | 13.831 | 0.487 | 6.727 | 0.932 |
| hsa-mir-130b-3p | 0.531 | 0.986 | 0.0633 | 589.924 | 313.372 | 1.718 | 0.306 |
| hsa-mir-19a-3p | 0.366 | 0.99 | 0.0683 | 6.781 | 2.481 | 2.396 | 0.464 |
| hsa-mir-455-5p | 0.392 | 0.999 | 0.109 | 45.652 | 17.885 | 2.127 | 0.437 |
| hsa-mir-218-5p | 0.154 | 1 | 0.128 | 4.394 | 0.676 | 2.248 | 0.733 |
| rno-mir-183-5p | 0.117 | 1 | 0.135 | 46.045 | 5.408 | 4.927 | 0.79 |
| hsa-mir-494-3p | 0.155 | 1 | 0.167 | 3.734 | 0.58 | 4.777 | 0.731 |
| hsa-mir-486-5p | 0.173 | 1 | 0.271 | 95.229 | 16.484 | 7.108 | 0.705 |
| hsa-mir-134-5p | 0.413 | 1 | 0.317 | 1.839 | 0.759 | 3.03 | 0.415 |
| hsa-mir-142-3p | 0.648 | 1 | 0.327 | 2.072 | 1.342 | 1.884 | 0.214 |
| hsa-mir-122-5p | 0.277 | 1 | 0.49 | 41.488 | 11.513 | 15.704 | 0.566 |
| hsa-mir-409-3p | 0.399 | 1 | 0.58 | 0.454 | 0.181 | 17.244 | 0.43 |
